# Discovery of 5-(5,5-Dimethylbutenolide-3-ethylidene)-2-amino-imidazolinone Derivatives as Fungicidal Agents

**DOI:** 10.3390/molecules200813740

**Published:** 2015-07-28

**Authors:** Bo Tang, Mingyan Yang, Yu Zhao, Lingqing Kong, Weiwei Wang, Mingan Wang

**Affiliations:** Department of Applied Chemistry, China Agricultural University, Beijing 100193, China; E-Mails: bs20143100240@cau.edu.cn (B.T.); yangmy@cau.edu.cn (M.Y.); lizhl@cau.edu.cn (Y.Z.); klq36211314@cau.edu.cn (L.K.); jay2hebe@cau.edu.cn (W.W.)

**Keywords:** butenolide, 2-aminoimidazolinone, synthesis, fungicidal activity

## Abstract

The novel fungicidal agents 5-(5,5-dimethylbutenolide-3-ethylidene)-2-amino-imidazolinone derivatives, were designed and synthesized in moderate to excellent yields in four steps by α-hydroxyketone and diketene as raw materials and characterized by HR-ESI-MS and ^1^H-NMR. The preliminary bioassay showed that some of these compounds, such as **4a**, **4e** and **5g** exhibit 94.9%, 92.8% and 81.4% inhibition rates against *Sclerotinia* *scleotiorum* at the concentration of 50 µg/mL, respectively. The EC_50_ values of compounds **4e** and **4i** were 4.14 and 3.27 µM against *Alternaria Solani*, and **5g** had EC_50_ value of 3.23 µM against *S. scleotiorum*. Compounds **4d** and **4g** displayed 98.0% and 97.8% control of spore germination against *Botrytis* *cinerea* at the concentration of 100 µg/mL, respectively.

## 1. Introduction

**Scheme 1:**
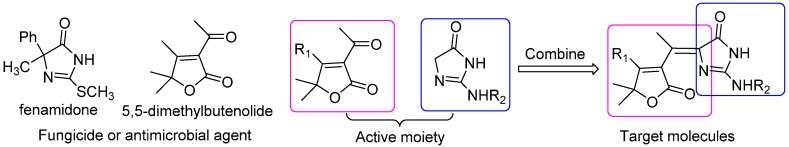
The design of target molecules based on the active groups.

**Scheme 2:**
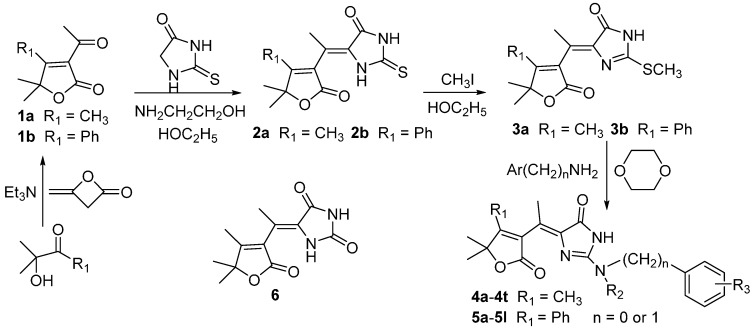
The synthetic route of 5-(butenolide-3-ethylidene)-2-aminoimidazolinone derivatives.

In recent years, various imidazolinone-containing compounds have been found in nature or synthesized and reported to display a wide range of biological activity such as fenamidone (Scheme 1) having antimicrobial and fungicidal activities [1,2,3,4,5,6,7,8,9]. Several 5-cyclohexylidene-2-aminoimidazolin-4-one derivatives have been prepared in our laboratory and showed significant fungicidal activities against several important phytopathgens [10,11]. Furthermore, the natural and unnatural butenolide derivatives including 3-acetyl-5,5-dimethylbutenolide (Scheme 1) exhibited interesting biological activities [12,13,14,15,16]. Some heterocyclic compounds derived from 3-acetyl-5,5-dimethylbutenolide were also reported, but their biological activities were not disclosed [17,18]. In order to continue our exploration of novel biologically active 2-aminoimidazolin-4-one heterocyclic compounds [10,11,19], butenolide and 2-aminoimidazolin-4-one were combined into a new class of 2-aminoimidazolin-4-one derivatives containing 5,5-dimethylbutenolide heterocycle derivatives as inhibitors of mitochondrial respiration, (Scheme 1 and Scheme 2) to improve their activities against the important agricultural diseases, which had not been published in literatures. In this paper, a series of 5-(5,5-dimethylbutenolide-3-ethylidene)-2-aminoimidazolinone derivatives were designed and synthesized, and their fungicidal activities and the structure-activity relationships were explored.

## 2. Results and Discussion

As indicated in the introduction, both imidazolinone and butenolide derivatives displayed a wide range of biological activity [1,2,3,4,5,6,7,8,9,12,13,14,15,16]. Based on these characters, we combined the butenolide and 2-aminoimidazolin-4-one into one molecule (Scheme 1) and synthesized the novel 2-aminoimidazolin-4-one derivatives containing butenolide heterocycles (**4**, **5**). As shown in Scheme 2, the intermediates **1a** and **1b** were synthesized by the reaction of α-hydroxyketone with diketene in 80% and 84% yields following the literature procedure [20], followed by reacting with thiohydantion catalysed by 2-aminoethanol to afford **2a** and **2b** in 63% and 67% yields, respectively [10,11]. Then methylations of **2a** and **2b** with methyl iodide were carried out at ambienent temperature to give the key intermediates **3a** and **3b** in 90% and 79% yields [19]. Finally, the intermediates **3a** and **3b** reacted with anilines or benzyl amines under reflux to produce the target compounds **4a**–**4t** and **5a**–**5l** in moderate to excellent yields. Interestingly, if acetic acid was used as solvent, compound **6** was isolated as the byproduct in 10%–40% yield when we first run the preparing reactions of **4a**–**4t** and fully characterized it by ^1^H-NMR, ^13^C-NMR and HR-ESI-MS. However, after changing from acetic acid to dioxane and adding oxalic acid to the reaction system, the byproduct was not observed. Moreover, purification of the final products was easier and the yields were also improved. So the preparation of compounds **5a**–**5l** selected the dioxane as solvent and added oxalic acid into the reaction system to effectively avoid the byproduct. In the ^1^H-NMR spectrum, the two methyl groups had the same chemical shifts in compounds **1a**, **2a**, **3a** and **4a**–**4t**, while the two methyl groups in compounds **1b**, **2b**, **3b** and **5a**–**5l** had different chemical shifts due to the orientation of phenyl group at 4-position in the butenolide moiety (see Supplementary Materials). The ^1^H-NMR spectra of **4a**–**4t** and **5a**–**5l** were similar to those compounds in the literatures [10,19].

Based on the data in Table 1, compounds **4** and **5** showed a broad-spectrum of fungicidal activities against these six tested phytopathgens. They were found to be particularly active against *Sclerotinia scleotiorum* and *Rhizoctonia* *solani*, for example, **4a**, **4e** and **5g** exhibited 94.9%, 92.8% (much higher than the commecialized carbendazim and nearly equal to chlorothalonil and fenamidone) and 81.4% inhibition rates against *S.* *scleotiorum*, **4d**, **4g**, **4i**, **4j**, **4m** and **4t** showed 72.9%, 72.9%, 72.1%, 75.7%, 72.0%, and 72.5% inhibition rates against *R. solani* at the concentration of 50 µg/mL, respectively.

**Table 1:** The fungicidal activities (inhibition rate, %) of compounds **4** and **5** at 50 µg/mL.

Compd.	n R_2_ R_3_	*S. scleotiorum*	*R. solani*	*A. solani*	*B. cinerea*	*P. capsici*	*F. graminearum*
**1a**		0.0	0.0	0.0	0.0	0.0	0.0
**1b**		0.0	0.0	0.0	0.0	0.0	0.0
**2a**		49.7	66.8	42.3	52.2	69.8	31.8
**2b**		3.3	7.7	1.5	36.2	59.3	3.9
**3a**		25.4	72.7	51.9	20.0	79.4	34.0
**3b**		0.0	18.6	0.0	16.8	34.2	10.6
**4a**	0 H H	94.9	68.4	50.9	60.5	24.0	21.1
**4b**	0 H 4-CH_3_	33.1	63.5	60.3	27.2	29.3	31.6
**4c**	0 H 4-F	19.1	59.6	63.2	31.8	16.7	38.2
**4d**	0 H 4-OCH_3_	32.3	72.9	60.8	75.3	15.5	38.6
**4e**	0 H 4-CF_3_	92.8	61.2	64.8	32.2	0.0	48.7
**4f**	0 CH_3_ H	31.7	57.6	53.9	16.5	36.1	0.0
**4g**	0 H 3-CF_3_	65.0	72.9	55.7	69.3	47.7	38.0
**4h**	0 H 2-CH_3_	16.5	56.4	37.0	36.2	15.7	0.0
**4i**	0 H 2-F	38.9	76.1	67.7	40.2	0.0	23.9
**4j**	0 H 2-OCH_3_	22.2	75.7	22.0	21.4	33.9	31.5
**4k**	0 H 2-Cl	18.7	44.6	44.6	45.5	41.8	37.4
**4l**	0 H 2,6-(CH_3_)_2_	30.8	65.3	45.9	29.1	2.1	39.2
**4m**	1 H H	52.2	72.0	53.9	48.4	23.0	22.9
**4n**	1 H 4-OCH_3_	36.9	66.5	39.0	28.6	0.0	51.3
**4o**	1 H 4-Cl	16.3	68.2	48.1	40.3	5.3	30.7
**4p**	1 H 4-F	21.5	48.5	45.4	39.2	33.6	38.5
**4q**	1 H 3-CF_3_	21.7	64.9	61.2	33.3	56.6	45.1
**4r**	1 H 2-F	15.4	59.5	41.2	31.6	27.6	23.5
**4s**	1 H 2-Cl	21.1	60.4	38.5	27.0	21.3	13.0
**4t**	1 H 2-OCH_3_	22.2	72.5	42.1	41.5	0.0	10.1
**5a**	0 H H	67.8	7.6	0.0	21.3	27.7	26.4
**5b**	0 H 4-CH_3_	29.4	10.9	11.6	25.1	13.3	22.0
**5c**	0 H 4-F	44.6	0.0	34.9	25.2	48.0	37.1
**5d**	0 H 4-OCH_3_	21.4	6.9	0.0	15.2	20.0	16.7
**5e**	0 H 4-CF_3_	58.1	11.4	31.4	38.6	50.4	24.1
**5f**	0 H 3-CF_3_	36.8	11.4	65.7	48.6	44.4	32.6
**5g**	0 H 2-F	81.4	12.9	14.4	26.3	45.1	59.9
**5h**	0 H 2-Cl	57.8	2.3	37.3	30.3	37.1	40.2
**5i**	0 H 2-OCH_3_	31.2	1.5	0.0	6.8	8.7	20.0
**5j**	1 H H	18.3	9.6	5.2	0.0	2.4	28.3
**5k**	1 H 4-Cl	29.2	0.0	24.4	12.6	13.3	15.4
**5l**	1 H 2-OCH_3_	30.0	6.9	3.0	8.5	0.0	27.7
**6**		41.7	46.1	70.7	40.9	4.2	31.6
Chlorothalonil		100	99.9	100	100	99.1	95.8
Carbendazim		81.0	100	100	4.2	34.7	100
Fenamidone		97.8	96.4	93.5	57.1	79.8	71.9

Comparing **2a** and **3a** with **1a**, it seems that the thiohydantoin ring was responsible for higher activity. Change from thione to methylthio made little contribution to the bioactivity against *R. solani* and *Phytophthora capsici*. Consequently, methylthio was replaced with phenylamino to obtain compound **4a**, which exhibited lower activity than **3a** against *R. solani* and much lower activity against *P. capsici*. Surprisingly, compound **4a** was found to have excellent activity (94.9%) on *S. scleotiorum*, which is much higher than the commecialized carbendazim and nearly equal to chlorothalonil and fenamidone. These results indicated that the introduction of phenylamino at the 2-position of the imidazolinone changed the activity spectrum. Inspired by this outstanding result, attention was turned to prepartion and evaluation of more analogous of **4a** by replacing substituents attached to the phenyl ring. The results indicated that with a strong electron-withdrawing group, such as trifluoromethyl (CF_3_) at the 4-positon (**4e**), almost equal activity against *S. scleotiorum* was noted. The introduction of CH_3_, F and OCH_3_ to any position resulted in the remarkable decrease in fungicidal activity. The compounds with substituents of 2-OCH_3_, 4-OCH_3_, 3-CF_3_ and 2-F on phenyl ring, however, exhibited better inhibition against *R. solani*, but still lower than three known products, which seemed that there was not much correlation between electronic effect and activity. Further optimization by replacing phenyl with benzyl led to much more decreased activities against all treated target phytopathgens.

As for R_1_ groups at 4-position of butenolide, the results indicated that the thiohydantoin **2a** and 2-methylthioimidazolinone **3a** exhibited inhibition against all tested phytopathgens, especially against *R. solani* and *P. capsici*. In contrast, **2b** and **3b** showed a significant decrease in activity against all six phytopathgens, indicating that R_1_ groups at 4-position of butenolide play a crucial role for regulating the bioactivity. This is probably due to steric hindrance of the phenyl group compared with methyl group. This conclusion was confirmed by comparison of the inhibition rates of **4a**–**4t** and corresponding **5a**–**5l**.

**Figure 1:**
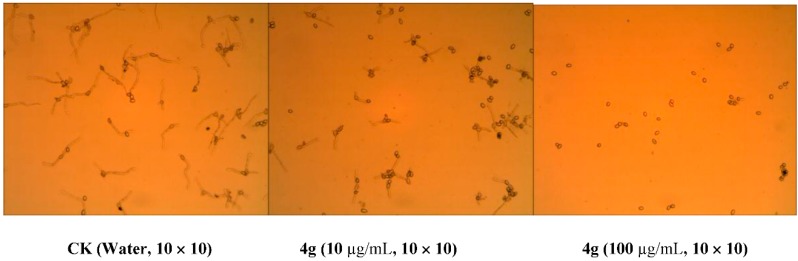
Inhibition of compound **4g** against spore germination of *B.* *cinerea*. The pictures were taken from the fungi growing in the glass plates with a microscope with Scopephoto software (version 3.6, Photoscope Inc., Arlington, VA, USA, 2009) and magnified by 10 × 10 times without stain. The fungi were grown in clear water (CK) or sample solution plus water (treated) at 25 ± 0.5 °C for 24 h. Color is from background.

The results above indicated that the aniline moiety should be optimized around compounds **4a** and **4e** and R_1_ prefers substituent with small size in the structure modification in the future. Based on the above results, the EC_50_ values were determined for these compounds with more than 65% inhibition rates. The equations for linear relationships of the logarithmic values of inhibition rates and the logarithmic values of concentrations were obtained with good coefficent γ^2^ as indicated in Table 2. The data in Table 2 indicated that **4e**, **4i** and **5g** have EC_50_ values less than 5.0 µM against *S. scleotiorum* and *A. Solani*, close or weaker than carbendazim and fenamidone, and the others have EC_50_ values more than 5.0 µM against all four phytopathgens. Among these compounds, most compounds did not exhibit the inhibition of spore germination against *S.* *scleotiorum*, *R. Solani* and *A. solani*, while compounds **4d** and **4g** demonstrated a siginificant control of spore germination against *B. cinerea* with 98.0% and 97.8% inhibition rates at the concentration of 100 µg/mL, respectively. The inhibition of spore germination against *B. cinerea* for **4g** was still observed even at a concentration of 10 µg/mL, as shown in Figure 1. The mode of action will be a topic of the future research. These results indicated that there is the possibility to improvement of fungicidal activities by modification of chemical structures.

**Table 2:** The EC_50_ values of compounds **4** and **5** against different phytopathgens.

Compd.	Fungi	Equation	Coefficent γ^2^	EC_50_ (µM)
**4a**	*S. scleotiorum*	Y = 4.151X − 16.812	0.966	268.58
**4e**	*S. scleotiorum*	Y = 2.181X − 7.968	0.990	220.54
**4g**	*S. scleotiorum*	Y = 2.236X − 9.418	0.950	14.73
**5g**	*S. scleotiorum*	Y = 0.902X − 3.382	0.978	3.23
Carbendazim	*S. scleotiorum*	Y = 1.143X − 4.821	0.981	4.12
**2a**	*R. solani*	Y = 2.993X − 12.458	0.949	120.78
**3a**	*R. solani*	Y = 2.136X − 8.651	0.972	38.25
**4a**	*R. solani*	Y = 3.052X − 12.407	0.956	218.48
**4d**	*R. solani*	Y = 2.152X − 8.824	0.953	24.18
**4g**	*R. solani*	Y = 2.170X − 8.084	0.971	149.00
**4i**	*R. solani*	Y = 1.797X − 6.847	0.963	48.73
**4j**	*R. solani*	Y = 8.084X − 36.222	0.972	4425.75
**4l**	*R. solani*	Y = 3.608X − 15.344	0.981	146.93
**4m**	*R. solani*	Y = 2.80X − 12.685	0.953	6.07
**4n**	*R. solani*	Y = 1.440X − 5.298	0.962	21.68
**4o**	*R. solani*	Y = 3.995X − 17.315	0.985	122.76
**4t**	*R. solani*	Y = 2.538X − 11.106	0.950	10.38
**4e**	*A. solani*	Y = 1.283X − 5.263	0.981	4.14
**4i**	*A. solani*	Y = 1.459X − 6.242	0.997	3.27
**6**	*A. solani*	Y = 1.263X − 4.926	0.978	9.86
Fenamidone	*A. solani*	Y = 0.802X − 3.709	0.973	0.90
**4d**	*B. cinerea*	Y = 2.127X − 8.363	0.957	52.49
**4g**	*B. cinerea*	Y = 1.514X − 5.267	0.986	51.02
Fenamidone	*B. cinerea*	Y = 1.329X − 4.712	0.963	38.92

## 3. Experimental Section

### 3.1. General Information

All reactions were performed with magnetic stirring. Unless otherwise stated, all reagents were purchased from commercial suppliers and used without further purification. Organic solutions were concentrated under reduced pressure using a rotary evaporator. Melting points were measured on a Yanagimoto apparatus (Yanagimoto MFG Co., Kyoto, Japan) and are uncorrected. ^1^H-NMR spectra were obtained on Bruker DPX 300 spectrometer (Bruker Biospin Co., Stuttgart, Germany) with CDCl_3_ or DMSO-*d*_6_ as a solvent and TMS as an internal standard. High resolution mass spectral analysis was performed on a LTQ Orbitrap instrument (ThermoFisher scientific Inc., Waltham, MA, USA).

### 3.2. Synthesis

#### 3.2.1. Synthesis of 3-Acetyl-4-methyl(phenyl)-5,5-dimethylbutenolide (**1a** and **1b**)

The synthesis of the intermediates **1a** and **1b** were carried out according to the literature protocols and their spectral data were identical with those in the references [18,20]. Compound **1a**, colorless solid, yield 80%, m.p. 50–51 °C, ^1^H-NMR (CDCl_3_, 300 MHz) δ: 2.47 (s, 3H), 2.28 (s, 3H), 1.42 (s, 6H). Compound **1b**, colorless solid, yield 84%, m.p. 76–77 °C, ^1^H-NMR (CDCl_3_, 300 MHz) δ: 7.48–7.46 (m, 3H), 7.25–7.22 (m, 2H), 2.39 (s, 3H), 1.56 (s, 3H).

#### 3.2.2. Synthesis of 4-Methyl(phenyl)-5,5-dimethyl-3-ethylidenebutenolide-5-thiohydantoin (**2a** and **2b**)

The synthesis of the intermediates **2a** and **2b** were performed following the processes in our previous paper [10,11] by reaction of **1a** and **1b** with thiohydantoin. Compound **2a**, yellow solid, yield 63%, m.p. 218–220 °C, ^1^H-NMR (DMSO-*d*_6_, 300 MHz) δ: 12.12 (s, 1H), 11.99 (s, 1H), 2.03 (s, 3H), 1.87 (s, 3H), 1.42 (s, 6H); HR-ESI-MS *m*/*z*: calcd for C_12_H_15_N_2_O_3_S [M + H]^+^ 267.0798; found, 267.0798. Compound **2b**, yellow solid, yield 67%, m.p. 223–225 °C, ^1^H-NMR (DMSO-*d*_6_, 300 MHz) δ: 12.20 (s, 1H), 11.99 (s,1H), 7.51–7.45 (m, 3H), 7.35–7.30 (m, 2H), 1.80 (s, 3H), 1.63 (s, 3H), 1.46 (s, 3H); HR-ESI-MS *m*/*z*: calcd for C_17_H_17_N_2_O_3_S [M + H]^+^ 329.0954; found, 329.0944.

#### 3.2.3. Synthesis of 4-Methyl(phenyl)-5,5-dimethyl-3-ethylidenebutenolide-5-(2-methylthioimidazo-4-one) (**3a** and **3b**)

The synthesis of the intermediates **3a** and **3b** were performed according to the methods in our previous paper [10,11] by reaction of **2a** and **2b** with methyl iodide. Compound **3a**, yellow solid, yield 90%, m.p. 168–170 °C, ^1^H-NMR (CDCl_3_, 300 MHz) δ: 10.23 (s, 1H), 2.61 (s, 3H), 2.37(s, 3H), 1.90 (s, 3H), 1.54 (s, 3H), 1.50 (s, 3H); HR-ESI-MS *m*/*z*: calcd for C_13_H_17_N_2_O_3_S [M + H]^+^ 281.0954; found, 281.0955. Compound **3b**, yellow solid, yield 79%, m.p. 204–206 °C, ^1^H-NMR (CDCl_3_, 300 MHz) δ: 10.16 (s, 1H), 7.42–7.37 (m, 3H), 7.27–7.23 (m, 2H), 2.57 (s, 3H), 2.08 (s, 3H), 1.68 (s, 3H), 1.59 (s, 3H); HR-ESI-MS *m*/*z*: calcd for C_17_H_17_N_2_O_3_S [M + H]^+^ 343.1111; found, 343.1102.

#### 3.2.4. General Procedure for the Synthesis of Compounds **4** and **5**

To a stirred solution of 1.0 mmol **3a**, or **3b** in 20 mL of acetic acid or oxalic acid and dioxane, the amines were added at ambient temperature and heated to reflux for 10–24 h. The reactions were monitored by TLC. After completion, the solvents were removed under reduced pressure. The residues were purified by silica gel chromatography using CH_2_Cl_2_/acetone as eluents to afford compounds **4** and **5**.

*5-(4,5,5-Trimethyl-3-ethylidenebutenolide)-2-phenylaminoimidazolinone* **4a**, white solid, yield 65%, m.p. 203–206 °C, ^1^H-NMR (DMSO-*d*_6_, 300 MHz) δ: 10.44–9.68 (m, 2H), 7.71–6.99 (m, 5H), 2.12 (s, 3H), 1.86 (s, 3H), 1.43 (s, 6H); HR-ESI-MS *m*/*z*: calcd for C_18_H_20_N_3_O_3_ [M + H]^+^ 326.1499; found, 326.1499.

*5-(4,5,5-Trimethyl-3-ethylidenebutenolide)-2-(4-methylphenyl)aminoimidazolinone* **4b**, white solid, yield 83%, m.p. 208–210 °C, ^1^H-NMR (DMSO-*d*_6_, 300 MHz) δ: 10.40–9.52 (m, 2H), 7.59–7.12 (m, 4H), 2.26 (s, 3H), 2.09 (s, 3H), 1.85 (s, 3H), 1.43 (s, 6H); HR-ESI-MS *m*/*z*: calcd for C_19_H_22_N_3_O_3_ [M + H]^+^ 340.1656; found, 340.1655.

*5-(4,5,5-Trimethyl-3-ethylidenebutenolide)-2-(4-flurophenyl)aminoimidazolinone* **4c**, white solid, yield 56%, m.p. 194–196 °C, ^1^H-NMR (DMSO-*d*_6_, 300 MHz) δ: 10.53–9.66 (m, 2H), 7.71–7.14 (m, 4H), 2.10 (s, 3H), 1.85 (s, 3H), 1.43 (s, 6H); HR-ESI-MS *m*/*z*: calcd for C_18_H_19_FN_3_O_3_ [M + H]^+^ 344.1405; found, 344.1405.

*5-(4,5,5-Trimethyl-3-ethylidenebutenolide)-2-(4-methoxyphenyl)aminoimidazolinone* **4d**, yellow solid, yield 87%, m.p. 151–154 °C, ^1^H-NMR (DMSO-*d*_6_, 300 MHz) δ: 10.60–9.61 (m, 2H), 7.54 (d, *J* *=* 7.5 Hz, 2H), 6.88 (d, *J =* 7.5 Hz, 2H), 3.74 (s, 3H), 2.31 (s, 3H), 1.91 (s, 3H), 1.44 (s, 6H); HR-ESI-MS *m*/*z*: calcd for C_19_H_22_N_3_O_4_ [M + H]^+^ 356.1605; found, 356.1605.

*5-(4,5,5-Trimethyl-3-ethylidenebutenolide)-2-(4-trifluromethylphenyl)aminoimidazolinone* **4e**, yellow solid, yield 59%, m.p. 203–206 °C, ^1^H-NMR (DMSO-*d*_6_, 300 MHz) δ: 10.73–10.06 (m, 2H), 8.00–7.66 (m, 4H), 2.12 (s, 3H), 1.86 (s, 3H), 1.43 (s, 6H); HR-ESI-MS *m*/*z*: calcd for C_19_H_19_F_3_N_3_O_3_ [M + H]^+^ 394.1373; found, 394.1374.

*N-Methyl-5-(4,5,5-trimethyl-3-ethylidenebutenolide)-2-phenylaminoimidazolinone* **4f**, white solid, yield 56%, m.p. 195–198 °C, ^1^H-NMR (DMSO-*d*_6_, 300 MHz) δ: 7.54–7.44 (m, 5H), 3.61 (s, 3H), 2.32 (s, 3H), 1.90 (s, 3H), 1.41 (s, 6H); HR-ESI-MS *m*/*z*: calcd for C_19_H_22_N_3_O_3_ [M + H]^+^ 340.1656; found, 340.1655.

*5-(4,5,5-Trimethyl-3-ethylidenebutenolide)-2-(3-trifluromethylphenyl)aminoimidazolinone* **4g**, yellow solid, yield 66%, m.p. 148–151 °C, ^1^H-NMR (DMSO-*d*_6_, 300 MHz) δ: 10.70–9.82 (m, 2H), 8.20–7.30 (m, 4H), 2.33 (s, 3H), 1.86 (s, 3H), 1.47 (s, 6H); HR-ESI-MS *m*/*z*: calcd for C_19_H_19_F_3_N_3_O_3_ [M + H]^+^ 394.1373; found, 394.1374.

*5-(4,5,5-Trimethyl-3-ethylidenebutenolide)-2-(2-methylphenyl)aminoimidazolinone* **4h**, white solid, yield 89%, m.p. 184–187 °C, ^1^H-NMR (DMSO-*d*_6_, 300 MHz) δ: 10.38–9.00 (m, 2H), 7.20–6.97 (m, 4H), 2.26 (s, 3H), 2.09 (s, 3H), 1.84 (s, 3H), 1.42 (s, 6H); HR-ESI-MS *m*/*z*: calcd for C_19_H_22_N_3_O_3_ [M + H]^+^ 340.1656; found, 340.1654.

*5-(4,5,5-Trimethyl-3-ethylidenebutenolide)-2-(2-flurophenyl)aminoimidazolinone* **4i**, white solid, yield 74%, m.p. 166–169 °C, ^1^H-NMR (DMSO-*d*_6_, 300 MHz) δ: 10.54–9.87 (m, 2H), 7.68–7.21 (m, 4H), 2.32 (s, 3H), 2.00 (s, 3H), 1.48 (s, 6H); HR-ESI-MS *m*/*z*: calcd for C_18_H_19_FN_3_O_3_ [M + H]^+^ 344.1405; found, 344.1406.

*5-(4,5,5-Trimethyl-3-ethylidenebutenolide)-2-(2-methoxyphenyl)aminoimidazolinone* **4j**, yellow solid, yield 58%, m.p. 154–156 °C, ^1^H-NMR (DMSO-*d*_6_, 300 MHz) δ: 10.41–9.65 (m, 2H), 7.07–6.90 (m, 4H), 3.88 (s, 3H), 2.09 (s, 3H), 1.92 (s, 3H), 1.50 (s, 6H); HR-ESI-MS *m*/*z*: calcd for C_19_H_22_N_3_O_4_ [M + H]^+^ 356.1605; found, 356.1606.

*5-(4,5,5-Trimethyl-3-ethylidenebutenolide)-2-(2-chlororophenyl)aminoimidazolinone* **4k**, white solid, yield 23%, m.p.165–168 °C, ^1^H-NMR (DMSO-*d*_6_, 300 MHz) δ: 10.41–9.92 (m, 2H), 7.44–7.00 (m, 4H), 2.09 (s, 3H), 1.85 (s, 3H), 1.41 (s, 6H); HR-ESI-MS *m*/*z*: calcd for C_18_H_19_C_l_N_3_O_3_ [M + H]^+^ 360.1110; found, 360.1110.

*5-(4,5,5-Trimethyl-3-ethylidenebutenolide)-2-(2,6-dimethylphenyl)aminoimidazolinone* **4l**, white solid, yield 76%, m.p. 240–243 °C, ^1^H-NMR (DMSO-*d*_6_, 300 MHz) δ: 10.41–9.19 (m, 2H), 7.12–6.95 (m, 3H), 2.11 (s, 6H), 1.84 (s, 3H), 1.44 (s, 6H); HR-ESI-MS *m*/*z*: calcd for C_19_H_22_N_3_O_3_ [M + H]^+^ 340.1656; found, 340.1654.

*5-(4,5,5-Trimethyl-3-ethylidenebutenolide)-2-benzylaminoimidazolinone* **4m**, white solid, yield 30%, m.p. 168–170 °C, ^1^H-NMR (DMSO-*d*_6_, 300 MHz) δ: 10.57–9.81 (m, 2H), 7.34–7.25 (m, 5H), 4.51 (brs, 2H), 1.99 (s, 3H), 1.80 (s, 3H), 1.39 (s, 6H); HR-ESI-MS *m*/*z*: calcd for C_19_H_22_N_3_O_3_ [M + H]^+^ 340.1656; found, 340.1655.

*5-(4,5,5-Trimethyl-3-ethylidenebutenolide)-2-(4-methoxybenzyl)aminoimidazolinone* **4n**, white solid, yield 56%, m.p. 145–148 °C, ^1^H-NMR (DMSO-*d*_6_, 300 MHz) δ: 10.57–9.81 (m, 2H), 7.34–7.25 (m, 5H), 4.51 (brs, 2H), 3.73 (s, 3H), 1.99 (s, 3H), 1.80 (s, 3H), 1.39 (s, 6H); HR-ESI-MS *m*/*z*: calcd for C_20_H_24_N_3_O_4_ [M + H]^+^ 370.1761; found, 370.1761.

*5-(4,5,5-Trimethyl-3-ethylidenebutenolide)-2-(4-chlrobenzyl)aminoimidazolinone* **4o**, white solid, yield 54%, m.p. 147–150 °C, ^1^H-NMR (DMSO-*d*_6_, 300 MHz) δ: 10.79–9.07 (m, 2H), 7.43–7.34 (m, 4H), 4.48 (brs, 2H), 1.98 (s, 3H), 1.81 (s, 3H), 1.40 (s, 6H); HR-ESI-MS *m*/*z*: calcd for C_19_H_21_C_l_N_3_O_3_ [M + H]^+^ 374.1266; found, 374.1267.

*5-(4,5,5-Trimethyl-3-ethylidenebutenolide)-2-(4-flurobenzyl)aminoimidazolinone* **4p**, yellow solid, yield 59%, m.p. 140–143 °C, ^1^H-NMR (DMSO-*d*_6_, 300 MHz) δ: 10.57–8.32 (m, 2H), 7.62–6.77 (m, 4H), 4.47 (brs, 2H), 1.99 (s, 3H), 1.81 (s, 3H), 1.40 (s, 6H); HR-ESI-MS *m*/*z*: calcd for C_19_H_21_FN_3_O_3_ [M + H]^+^ 358.1562; found, 358.1561.

*5-(4,5,5-Trimethyl-3-ethylidenebutenolide)-2-(3-trifluromethylbenzyl)aminoimidazolinone* **4q**, yellow solid, yield 48%, m.p. 127–130 °C, ^1^H-NMR (DMSO-*d*_6_, 300 MHz) δ: 10.84–9.15 (m, 2H), 7.72–7.48 (m, 4H), 4.58 (brs, 2H), 1.97 (s, 3H), 1.81 (s, 3H), 1.44 (s, 6H); HR-ESI-MS *m*/*z*: calcd for C_20_H_21_F_3_N_3_O_3_ [M + H]^+^ 408.1530; found, 408.1528.

*5-(4,5,5-Trimethyl-3-ethylidenebutenolide)-2-(2-flurobenzyl)aminoimidazolinone* **4r**, yellow solid, yield 81%, m.p. 227–230 °C, ^1^H-NMR (DMSO-*d*_6_, 300 MHz) δ: 10.51–9.84 (m, 2H), 7.45–7.18 (m, 4H), 4.52 (brs, 2H), 1.99 (s, 3H), 1.82 (s, 3H), 1.41 (s, 6H); HR-ESI-MS *m*/*z*: calcd for C_19_H_21_FN_3_O_3_ [M + H]^+^ 358.1562; found, 358.1562.

*5-(4,5,5-Trimethyl-3-ethylidenebutenolide)-2-(2-chlorobenzyl)aminoimidazolinone* **4s**, white solid, yield 70%, m.p. 164–166 °C, ^1^H-NMR (DMSO-*d*_6_, 300 MHz) δ: 10.41–9.92 (m, 2H), 7.53–7.38 (m, 4H), 4.71 (brs, 2H), 2.32 (s, 3H), 2.01 (s, 3H), 1.44 (s, 6H); HR-ESI-MS *m*/*z*: calcd for C_19_H_21_C_l_N_3_O_3_ [M + H]^+^ 374.1266; found, 374.1267.

*5-(4,5,5-Trimethyl-3-ethylidenebutenolide)-2-(2-methoxybenzyl)aminoimidazolinone* **4t**, yellow solid, yield 34%, m.p. 120–123 °C, ^1^H-NMR (DMSO-*d*_6_, 300 MHz) δ: 10.40–8.83 (m, 2H), 7.30–6.91 (m, 4H), 4.46 (brs, 2H), 3.83 (s, 3H), 1.96 (s, 3H), 1.81 (s, 3H), 1.44 (s, 6H); HR-ESI-MS *m*/*z*: calcd for C_20_H_24_N_3_O_4_ [M + H]^+^ 370.1761; found, 370.1758.

*5-(4-Phenyl-5,5-dimethyl-3-ethylidenebutenolide)-2-phenylaminoimidazolinone* **5a**, yellow solid, yield 44%, m.p. 167–170 °C, ^1^H-NMR (DMSO-*d*_6_, 300 MHz) δ: 10.52–9.34 (m, 2H), 7.68–6.99 (m, 10H), 1.88 (s, 3H), 1.69 (s, 3H), 1.48 (s, 3H); HR-ESI-MS *m*/*z*: calcd for C_23_H_22_N_3_O_3_ [M + H]^+^ 388.1656; found, 388.1644.

*5-(4-Phenyl-5,5-dimethyl-3-ethylidenebutenolide)-2-(4-methylphenyl)aminoimidazolinone* **5b**, yellow solid, yield 60%, m.p. 185–188 °C, ^1^H-NMR (DMSO-*d*_6_, 300 MHz) δ: 10.47–9.32 (m, 2H), 7.47–7.04 (m, 9H), 2.25 (s, 3H), 1.87 (s, 3H), 1.61 (s, 3H), 1.47 (s, 3H); HR-ESI-MS *m*/*z*: calcd for C_24_H_24_N_3_O_3_ [M + H]^+^ 402.1812; found, 402.1801.

*5-(4-Phenyl-5,5-dimethyl-3-ethylidenebutenolide)-2-(4-flurophenyl)aminoimidazolinone* **5c**, yellow solid, yield 47%, m.p. 164–167 °C, ^1^H-NMR (DMSO-*d*_6_, 300 MHz) δ: 10.61–9.37 (m, 2H), 7.71–7.08 (m, 9H), 1.87 (s, 3H), 1.61 (s, 3H), 1.48 (s, 3H); HR-ESI-MS *m*/*z*: calcd for C_23_H_21_FN_3_O_3_ [M + H]^+^ 406.1561; found, 406.1549.

*5-(4-Phenyl-5,5-dimethyl-3-ethylidenebutenolide)-2-(4-methoxyphenyl)aminoimidazolinone* **5d**, yellow solid, yield 82%, m.p. 217–219 °C, ^1^H-NMR (DMSO-*d*_6_, 300 MHz) δ: 10.44–9.21 (m, 2H), 7.58–6.85 (m, 9H), 3.74 (s, 3H), 1.85 (s, 3H), 1.61 (s, 3H), 1.47 (s, 3H); HR-ESI-MS *m*/*z*: calcd for C_24_H_24_N_3_O_4_ [M + H]^+^ 418.1761; found, 418.1749.

*5-(4-Phenyl-5,5-dimethyl-3-ethylidenebutenolide)-2-(4-trifluromethylphenyl)aminoimidazolinone* **5e**, white solid, yield 86%, m.p. 190–193 °C, ^1^H-NMR (DMSO-*d*_6_, 300 MHz) δ: 10.45–9.35 (m, 2H), 7.97–7.30 (m, 9H), 1.89 (s, 3H), 1.62 (s, 3H), 1.48 (s, 3H); HR-ESI-MS *m*/*z*: calcd for C_24_H_21_F_3_N_3_O_3_ [M + H]^+^ 456.1530; found, 456.1518.

*5-(4-Phenyl-5,5-dimethyl-3-ethylidenebutenolide)-2-(3-trifluromethylphenyl)aminoimidazolinone* **5f**, white solid, yield 77%, m.p. 169–172 °C, ^1^H-NMR (DMSO-*d*_6_, 300 MHz) δ: 10.77–9.57 (m, 2H), 8.23–7.32 (m, 9H), 1.90 (s, 3H), 1.59 (s, 3H), 1.49 (s, 3H); HR-ESI-MS *m*/*z*: calcd for C_24_H_21_F_3_N_3_O_3_ [M + H]^+^ 456.1530; found, 456.1517.

*5-(4-Phenyl-5,5-dimethyl-3-ethylidenebutenolide)-2-(2-flurophenyl)aminoimidazolinone* **5g**, yellow solid, yield 32%, m.p. 158–161 °C, ^1^H-NMR (DMSO-*d*_6_, 300 MHz) δ: 10.51–9.22 (m, 2H), 7.62–7.04 (m, 9H), 1.80 (s, 3H), 1.61 (s, 3H), 1.45 (s, 3H); HR-ESI-MS *m*/*z*: calcd for C_23_H_21_FN_3_O_3_ [M + H]^+^ 406.1561; found, 406.1549.

*5-(4-Phenyl-5,5-dimethyl-3-ethylidenebutenolide)-2-(2-chlorophenyl)aminoimidazolinone* **5h**, yellow solid, yield 32%, m.p. 158–161 °C, ^1^H-NMR (DMSO-*d*_6_, 300 MHz) δ: 10.73–9.48 (m, 2H), 7.50–7.00 (m, 9H), 1.91 (s, 3H), 1.60 (s, 3H), 1.44 (s, 3H); HR-ESI-MS *m*/*z*: calcd for C_23_H_21_ClN_3_O_3_ [M + H]^+^ 406.1561; found, 406.1549.

*5-(4-Phenyl-5,5-dimethyl-3-ethylidenebutenolide)-2-(2-methoxyphenyl)aminoimidazolinone* **5i**, yellow solid, yield 84%, m.p. 236–239 °C, ^1^H-NMR (DMSO-*d*_6_, 300 MHz) δ: 10.18–9.41 (m, 2H), 7.46–6.94 (m, 9H), 3.88 (s, 3H), 1.89 (s, 3H), 1.61 (s, 3H), 1.47 (s, 3H); HR-ESI-MS *m*/*z*: calcd for C_24_H_24_N_3_O_4_ [M + H]^+^ 418.1761; found, 418.1748.

*5-(4-Phenyl-5,5-dimethyl-3-ethylidenebutenolide)-2-benzylaminoimidazolinone* **5j**, white solid, yield 55%, m.p. 128–131 °C, ^1^H-NMR (DMSO-*d*_6_, 300 MHz) δ: 10.60–9.33 (m, 2H), 7.45–7.27 (m, 10H), 4.51 (brs, 2H), 1.80 (s, 3H), 1.59 (s, 3H), 1.44 (s, 3H); HR-ESI-MS *m*/*z*: calcd for C_24_H_24_N_3_O_3_ [M + H]^+^ 402.1812; found, 402.1801.

*5-(4-Phenyl-5,5-dimethyl-3-ethylidenebutenolide)-2-(4-chlorobenzyl)aminoimidazolinone* **5k**, yellow solid, yield 67%, m.p. 140–143 °C, ^1^H-NMR (DMSO-*d*_6_, 300 MHz) δ: 10.67–9.40 (m, 2H), 7.46–7.17 (m, 9H), 4.46 (brs, 2H), 1.74 (s, 3H), 1.59 (s, 3H), 1.44 (s, 3H); HR-ESI-MS *m*/*z*: calcd for C_24_H_23_ClN_3_O_3_ [M + H]^+^ 436.1422; found, 436.1412.

*5-(4-Phenyl-5,5-dimethyl-3-ethylidenebutenolide)-2-(2-methoxybenzyl)aminoimidazolinone* **5l**, yellow solid, yield 67%, m.p. 116–119 °C, ^1^H-NMR (DMSO-*d*_6_, 300 MHz) δ: 10.18–9.41 (m, 2H), 7.46–6.94 (m, 9H), 3.88 (s, 3H), 1.89 (s, 3H), 1.59 (s, 3H), 1.44 (s, 3H); HR-ESI-MS *m*/*z*: calcd for C_25_H_26_N_3_O_4_ [M + H]^+^ 432.1918; found, 432.1916.

*4,5,5-Trimethyl-3-ethylidenebutenolide-5-hydantoin* **6**, white solid, yields 10%–40% isolated from the mixtures of preparing reactions **4a** to **4t** as byproducts, m.p. 230–232 °C, ^1^H-NMR (DMSO-*d*_6_, 300 MHz) δ: 10.98 (s, 1H), 10.26 (s, 1H), 1.90 (s, 3H), 1.85 (s, 3H), 1.41 (s, 6H); ^1^^3^C-NMR (DMSO-*d*_6_, 75 MHz) δ: 170.02, 167.04, 162.88, 154.54, 19.21, 124.00, 117.70, 85.83, 24.19, 24.09, 17.93, 11.73; HR-ESI-MS *m*/*z*: calcd for C_12_H_15_N_2_O_4_ [M + H]^+^ 251.1026; found, 251.1026.

### 3.3. Bioassay of Fungicidal Activity

The preliminary fungicidal activities of compounds **4**–**6** against *S. scleotiorum*, *R. solani*, *P. capsici*, *A. solani*, *B.* *cinerea* and *F.* *graminearum* were evaluated using methods in the references [21,22,23] by the mycelium growth rate and spore germination tests [24,25]. The cultures were incubated at 25 ± 0.5 °C. Three replicates were performed and the mean measurements were calculated from the three replicates. The EC_50_ values were determined from the inhibition rates of five different concentrations based on the statistics method for the compounds which had more than 65% inhibition rates. Chlorothalonil, carbendazim and fenamidone were used as positive controls in the mycelium growth rate test, while pure water was used as blank control in the spore germination test.

## 4. Conclusions

The novel 5-(5,5-dimethylbutenolide-3-ethylidene)-2-aminoimidazolinone derivatives were designed and synthesized in moderate to excellent yields in four steps by α-hydroxyketone and diketene as starting materials. The products were characterized by HR-ESI-MS and ^1^H-NMR. The preliminary bioassay showed that some of these compounds, such as **4a**, **4e** and **5g** exhibit 94.9%, 92.8% and 81.4% inhibition rates against *S.* *scleotiorum*, respectively. The EC_50_ values of compounds **4e** and **4i** were 4.14 and 3.27 µM against *A. Solani*, while **5g** had EC_50_ value of 3.23 µM against *S.* *scleotiorum*. Compounds **4d** and **4g** had 98.0% and 97.8% inhibition rates of spore germination against *B.* *cinerea* at the concentration of 100 µg/mL, respectively. Further syntheses and structure optimization studies are in progress in our laboratory.

## Supplementary Materials

Supplementary materials can be accessed at: http://www.mdpi.com/1420-3049/20/08/13740/s1.

## References

[1] Wang Y., Xie H., Pan Y.R., Ding M.W. (2014). Facile synthesis of 4-arylidene-1*H*-imidazol-5(4*H*)-ones by an Ugi-Aza-Wittig sequences. Synthesis.

[2] Revanasiddappa B.C., Kalsi J., Jisha M.S., Jose N., Varghese S.S. (2013). Synthesis and biological evaluation of some novel imidazolinone derivatives. Indian J. Heterocycl. Chem..

[3] Miqdad O.A., Abunada N.M., Hassaneen H.M., Abu Samaha A.M. (2011). Synthesis and biological activity evaluation of some new heterocyclic spirocompounds with imidazolinone and pyrazoline moieties. Int. J. Chem..

[4] Patel J.B., Desai V.A. (2011). Discovery of new imidazolinone derivatives as potential microbial agents. Int. J. Drug Des. Discov..

[5] Ivanna S., Dmytro A., Ewa S., Katarzyna K.K., Borys Z., Olexandr V., Andrezj G., Roman L. (2010). Synthesis of 5-arylidene-2-amino-4-azolones and evaluation of their anticancer activity. Bioorg. Med. Chem..

[6] Huang X.B., Wu D.Z., Ding J.C., Zhang X.C., Liu M.C., Wu H.Y., Su W.K. (2010). Synthesis and biological activities of new chiral imidazolone derivatives. Phosphorus Sulfur Silicon.

[7] Huang X.B., Liu J.Z., Yang F.L., Ding M.W. (2007). Synthesis and properties of novel imidazolone derivatives containing a sulfur atom. Phosphorus Sulfur Silicon.

[8] Paola V., Athina G., Kitka A., Matteo I., Franca Z. (2006). Synthesis and antimicrobial activity of novel 2-thiozolyimino-5-arylidene-2-amino-4-thiazolidinones. Bioorg. Med. Chem..

[9] Travert N., Al Mourabit A. (2004). A likely biogenetic gateway linking 2-aminoimidazolinone metabolites of sponges to proline: Spontaneous oxidative conversion of the pyrrole-proline guanidine pseudo-peptide to dispacamide A. J. Am. Chem. Soc..

[10] Lei J.P., Han J.T., Xu Z.H., Dong H.B., Wang M.A. (2012). Synthesis and fungicidal activity of 5-cyclohexylidene-2-aminoimidazolin-4-one derivatives. Chin. J. Org. Chem..

[11] Lei J.P., Dong H.B., Tang B., Wang M.A. (2012). Synthesis and fungicidal activity of 5-(2-ethylcyclohexylidene)-2-aminoimidazolin-4-one derivatives. Chin. J. Pestic. Sci..

[12] Wang W.H., Kim H.Y., Nam S., Rho B.J., Kang H. (2012). Antibacterial butenolides from the Korean tunicate *Pseudodistoma antinboja*. J. Nat. Prod..

[13] Centko R.M., Ramón-García S., Taylor T., Patrick B.O., Thompson C.J., Miao V.P., Andersen R.J. (2012). Ramariolides A–D, antimycobacterial butenolides isolated from the mushroom *Ramaria cystidiophora*. J. Nat. Prod..

[14] Schulz D., Ohlendorf B., Zinecker H., Schmaljohann R., Imhoff J.F. (2011). Eutypoids B–E produced by a *Penicillium* sp. strain from the North Sea. J. Nat. Prod..

[15] Vinale F., Marra R., Scala F., Ghisalberti E.L., Lorito M., Sivasithamparam K. (2006). Major secondary metabolites produced by two commercial *Trichoderma* strains active against different phytopathogens. Lett. Appl. Microbiol..

[16] Wangteeraprasert R., Lipipun V., Gunaratnam M., Neidle S., Gibbons S., Likhitwitayawuid K. (2012). Bioactive compounds from *Carissa spinarum*. Phytother. Res..

[17] Leite L., Jansone D., Veveris M., Cirule H., Popelis J., Melikyan G., Avetisyan A., Lukevics E. (1999). Vasodilating and antiarrhythmic activity of heteryl lactones. Eur. J. Med. Chem..

[18] Melikyan G.S., Hovhannisyan A.A., Hayotsyan S.S. (2012). Synthesis of some heterocyclic compounds from enamines of 3-acetylfuran-2(5*H*)-ones. Synth. Commun..

[19] Kiec-Kononowicz K., Szymanska E. (2002). Antimycobacterial activity of 5-arylidene derivatives of hydantoin. Farmaco.

[20] Lacey R.N. (1954). Derivatives of acetoacetic acid. Part IV. A new route to α-acetyltetronic acids. J. Chem. Soc..

[21] Han J.T., Dong H.B., Xu Z.H., Wang J.M., Wang M.A. (2013). Synthesis and Activity of Novel Acylthiourea with Hydantoin. Int. J. Mol. Sci..

[22] Han J.T., Dong H.B., Xu Z.H., Lei J.P., Wang M.A. (2013). Facile synthesis of 5-arylidene thiohydantoin by sequential sulfonylation/desulfination reaction. Int. J. Mol. Sci..

[23] Liu B., Han J.T., Tang B., Wang M.A. (2014). Synthesis and Fungicidal Activity of d-Ribose and d-Xylose with Hydantion. Chin. J. Org. Chem..

[24] Chen N.C. (1991). The Bioassay Technologies for Pesticides.

[25] Chen W.Y. (2007). The Research & Development of New Pesticide—Methods & Progress.

