# Supplementary material for: Discovery of 5-(5,5-Dimethylbutenolide-3-ethylidene)-2-amino-imidazolinone Derivatives as Fungicidal Agents
Source: Molecules. 2015 Jul 28;20(8):13740–52. doi: 10.3390/molecules200813740 (PMC6332411; doi:10.3390/molecules200813740)
Supplement: Supplementary file 1 [file molecules-20-13740-s001.pdf]

Supplementary Materials

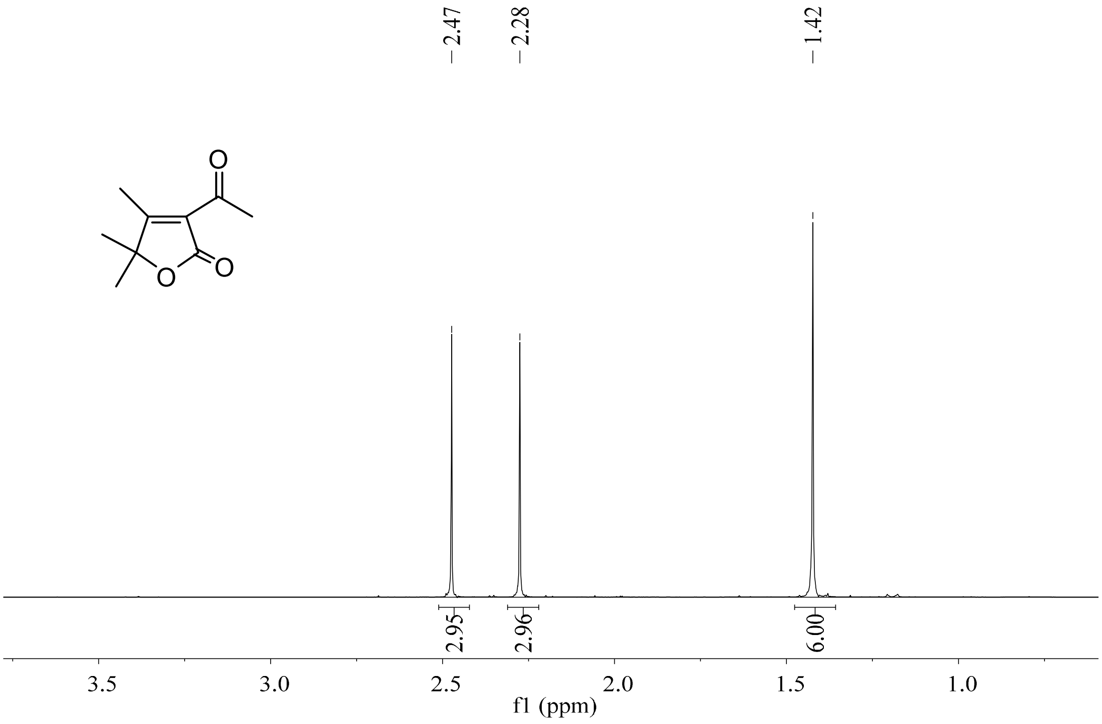

Figure S1. ^{1}H-NMR of **1a**.

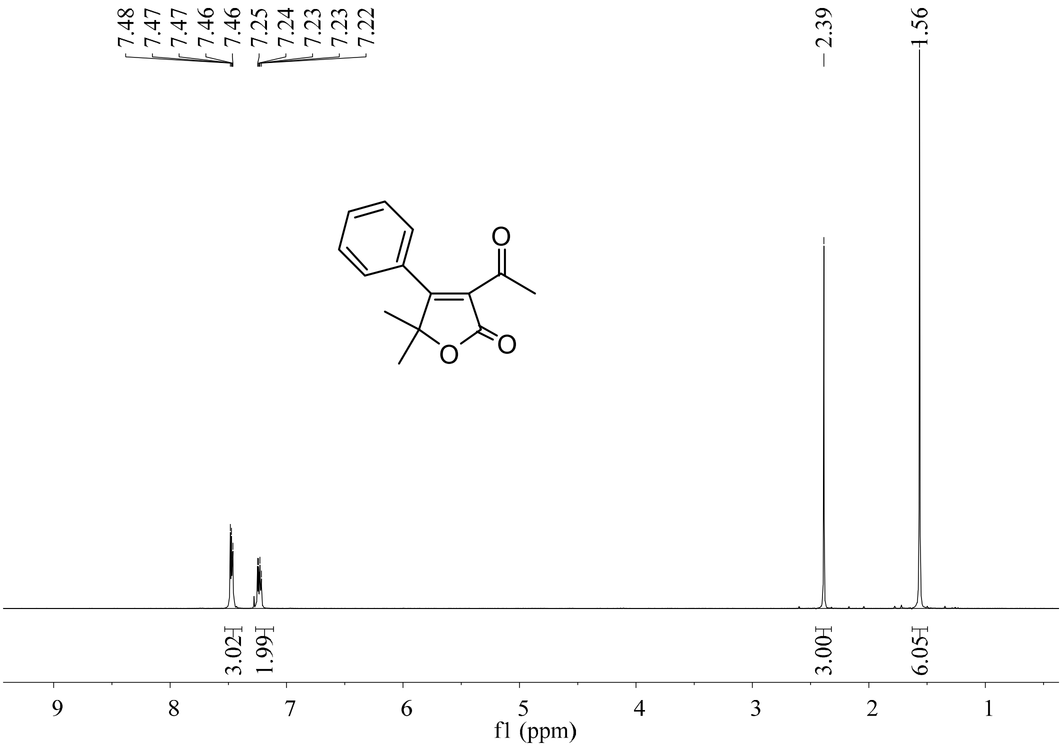

Figure S2. ^{1}H-NMR of **1b**.

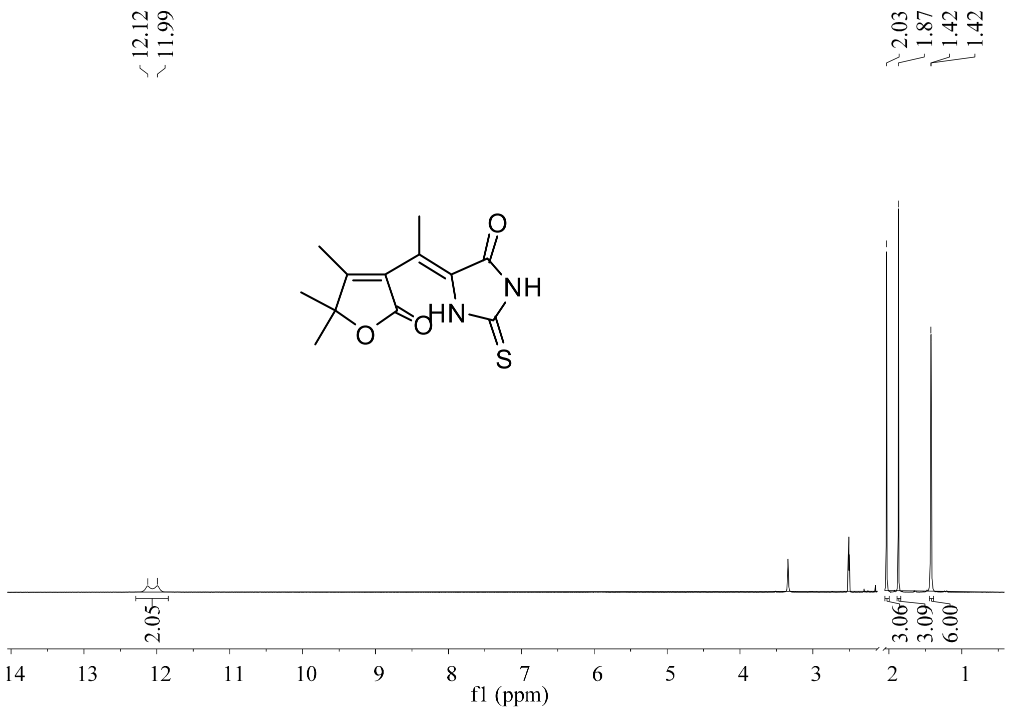

Figure S3. ¹H-NMR of **2a**.

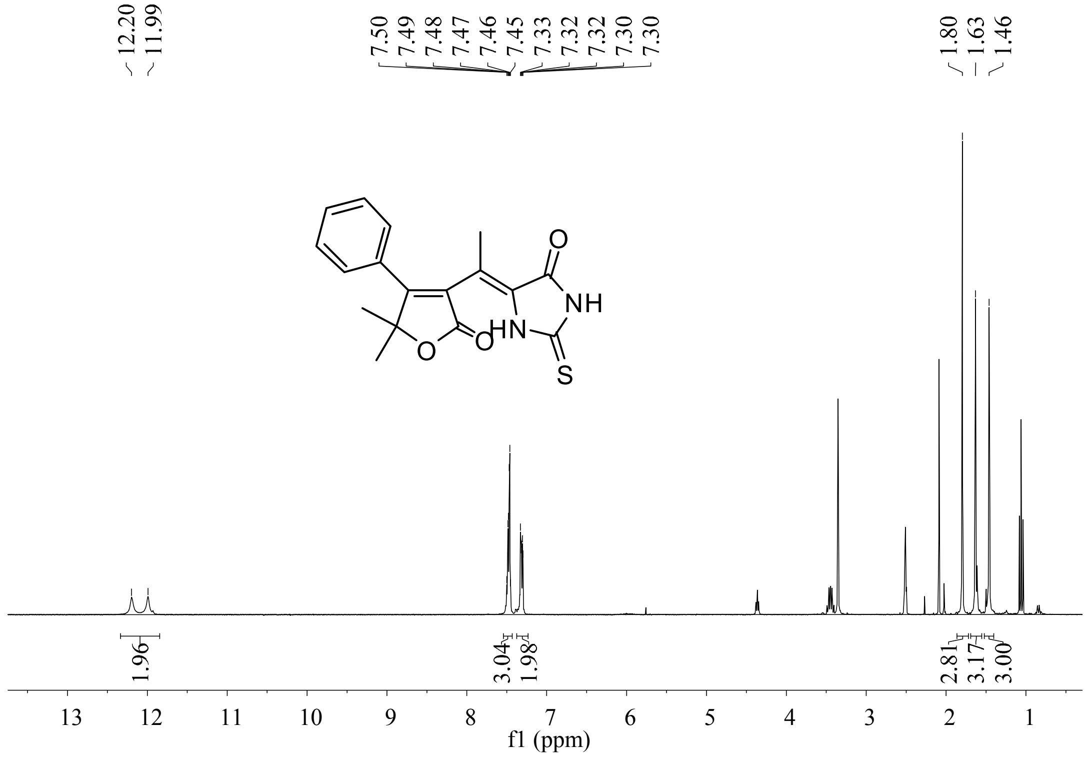

Figure S4. ¹H-NMR of **2b**.

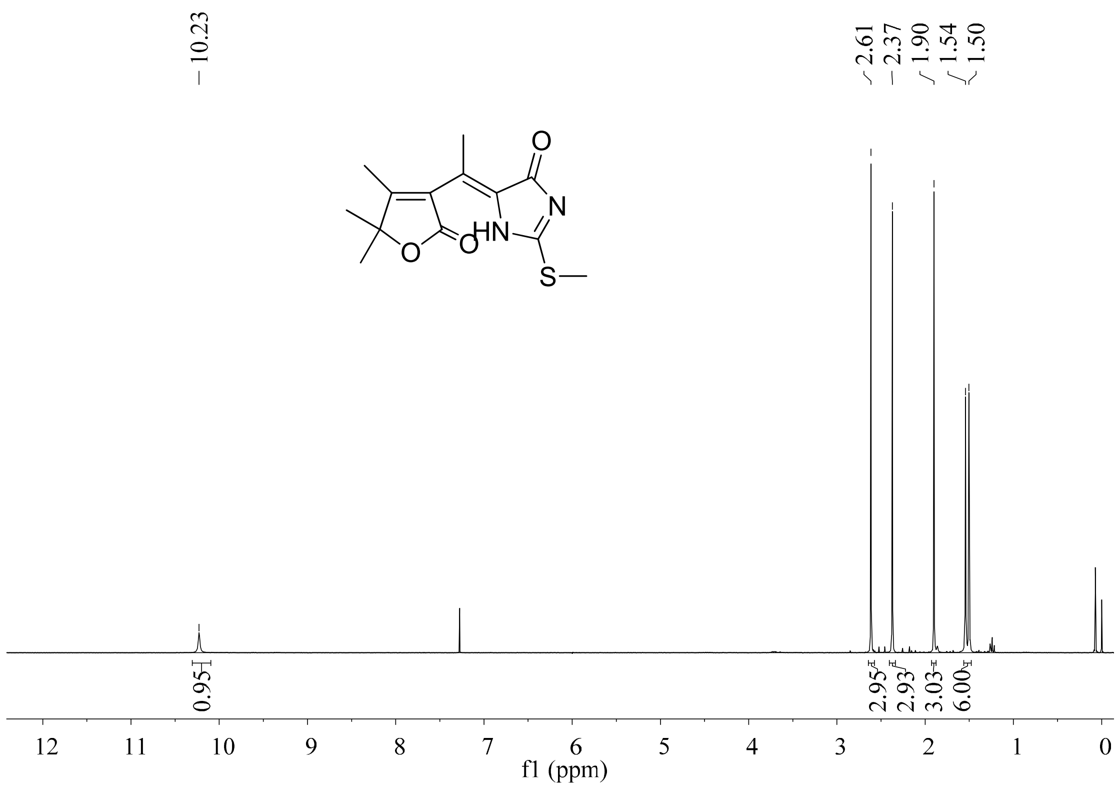

Figure S5. ^{1}H-NMR of **3a**.

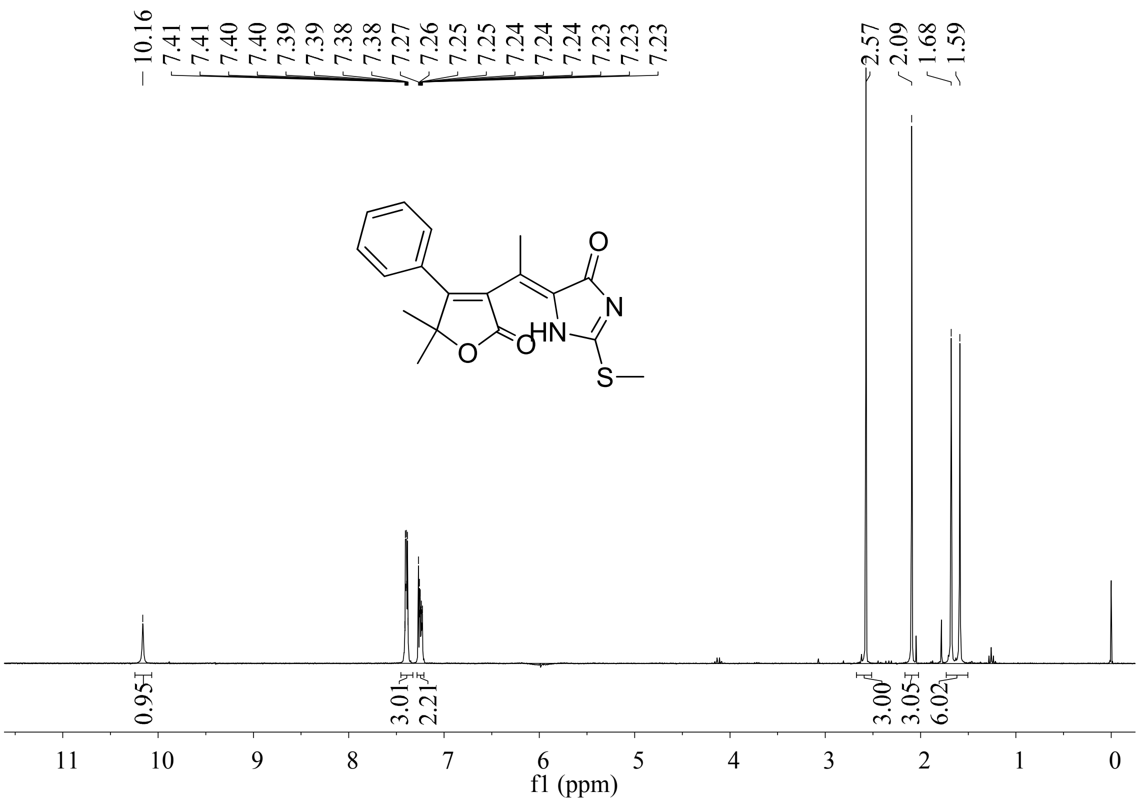

Figure S6. ^{1}H-NMR of **3b**.

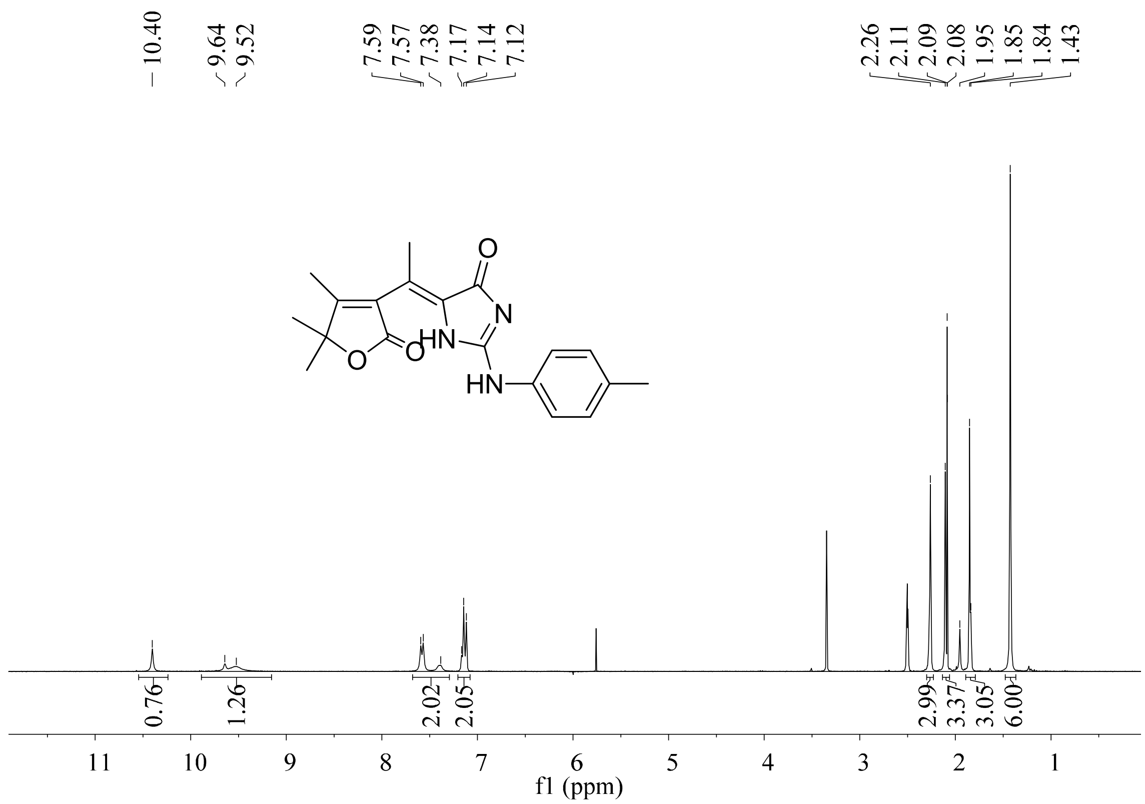

Figure S7. ^{1}H-NMR of **4b**.

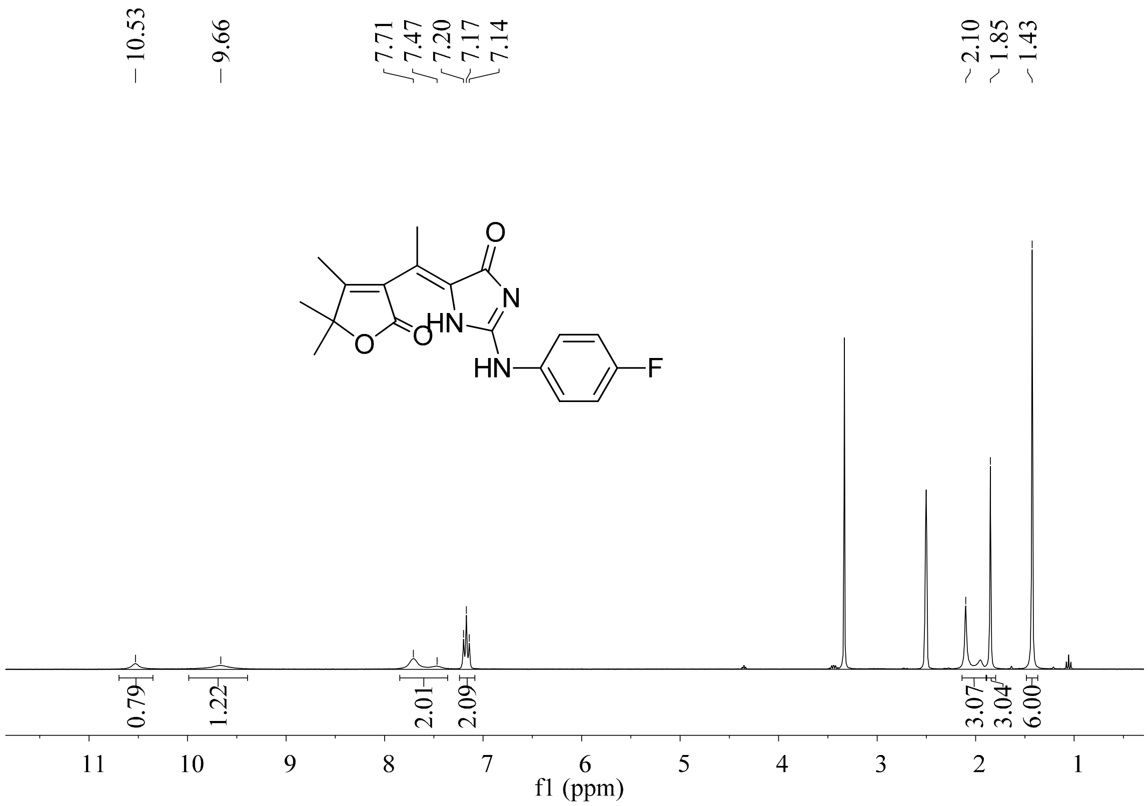

Figure S8. ^{1}H-NMR of **4c**.

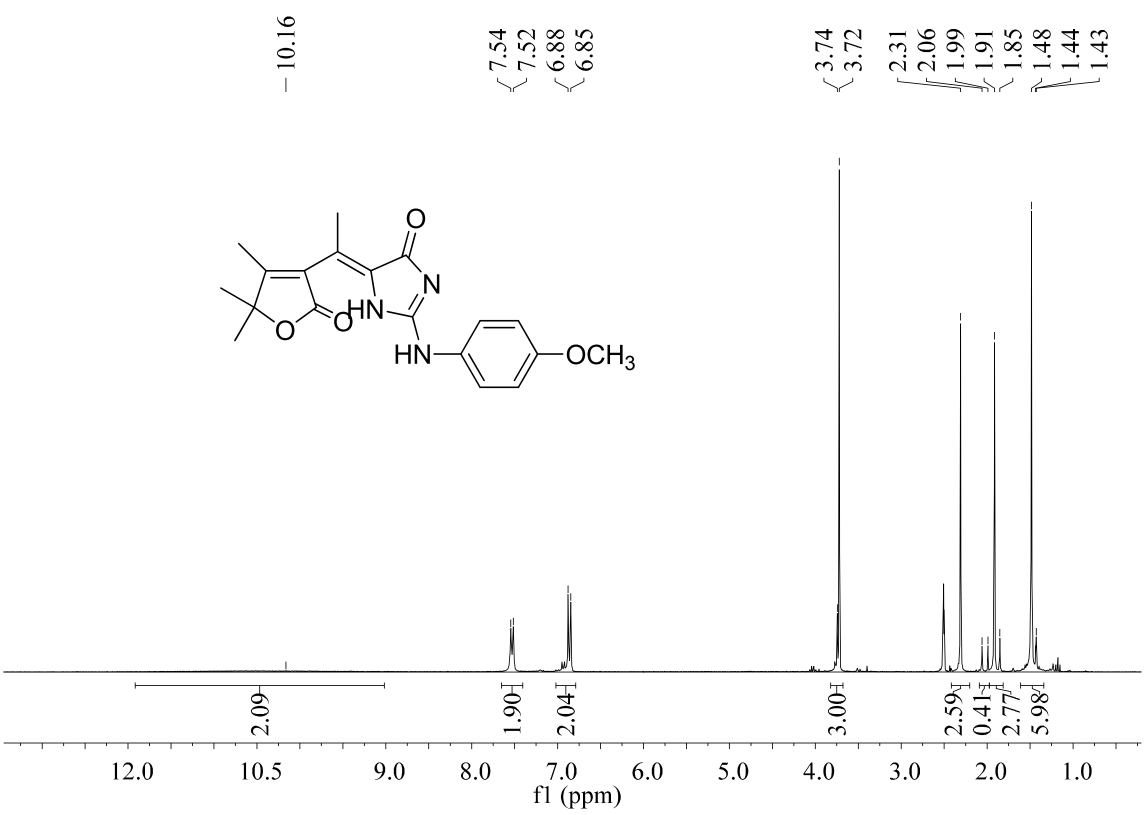

Figure S9. ^{1}H-NMR of **4d**.

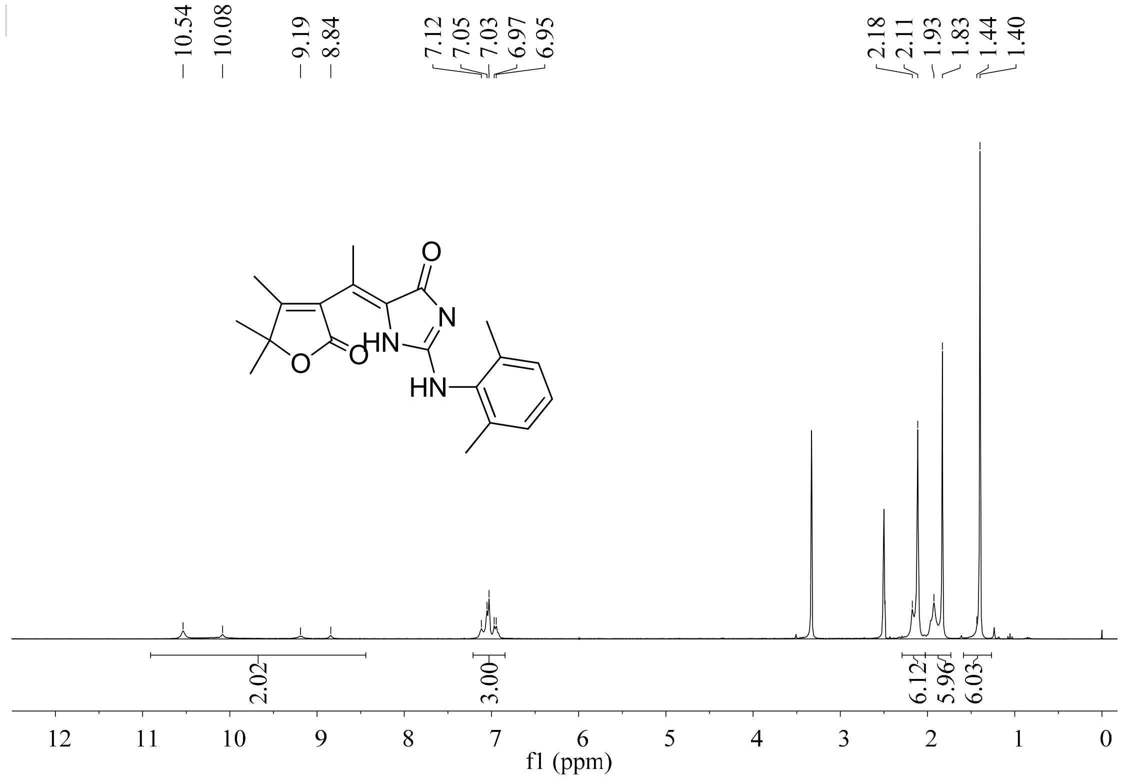

Figure S10. ^{1}H-NMR of **4l**.

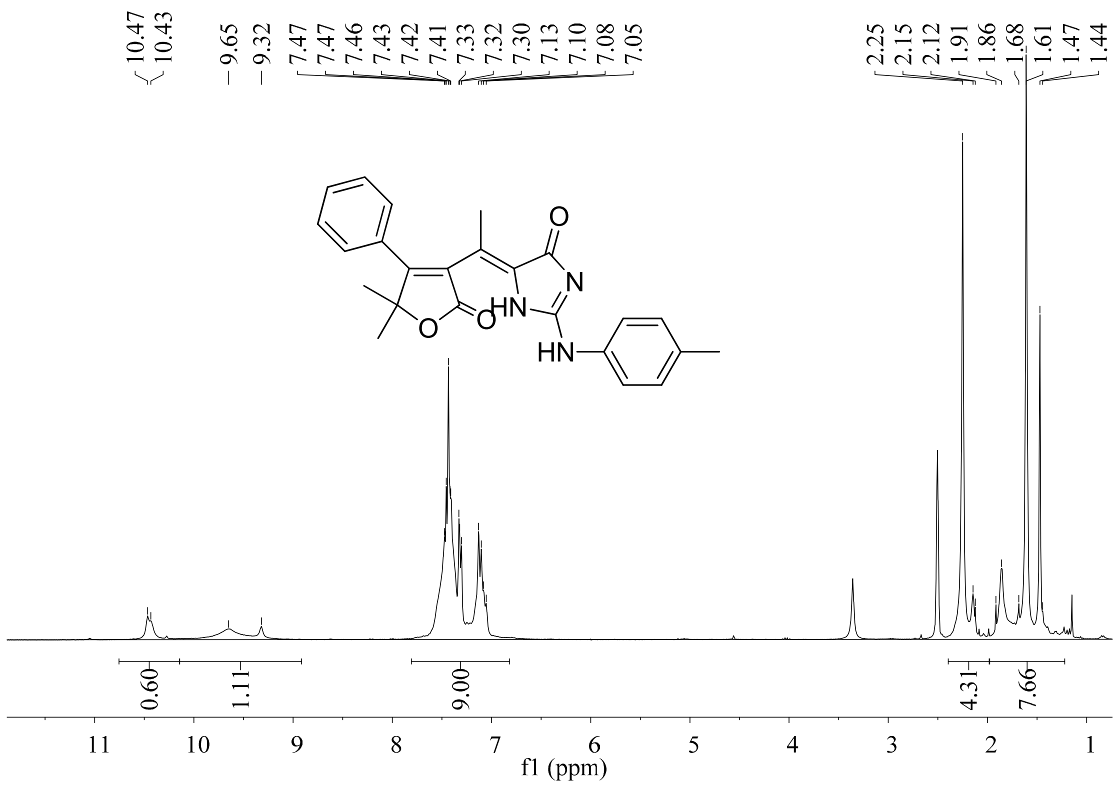

Figure S11. ^{1}H-NMR of **5b**.

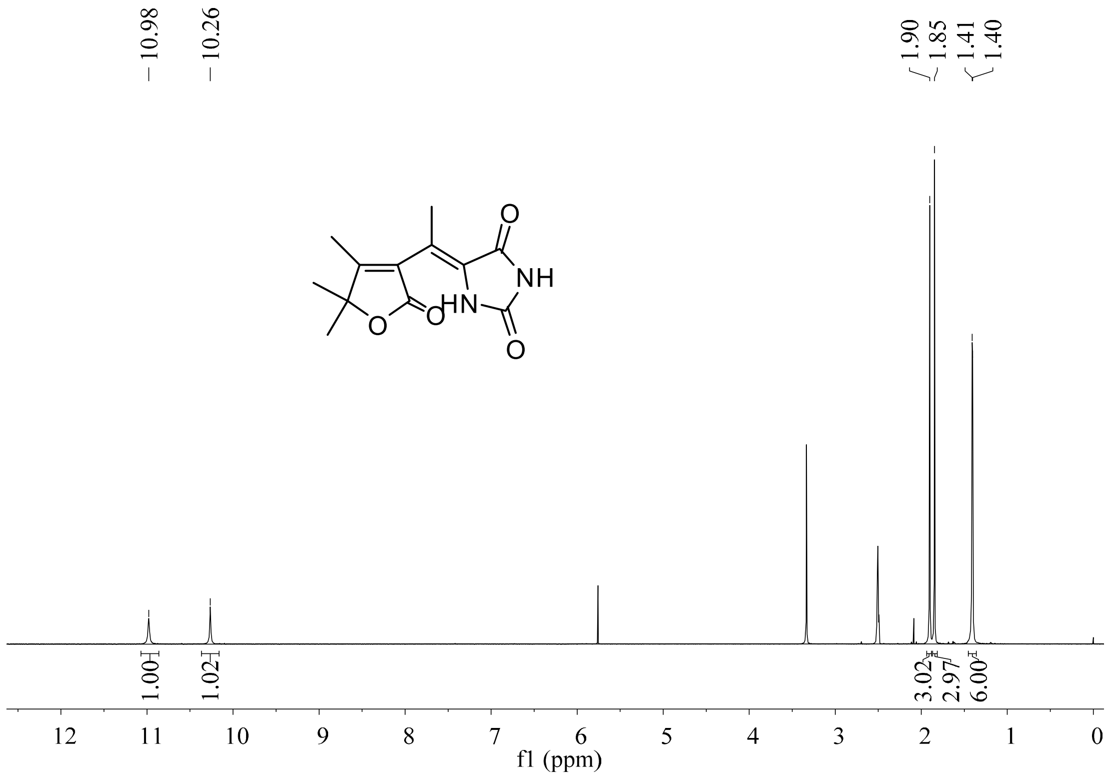

Figure S12. ^{1}H-NMR of **6**.

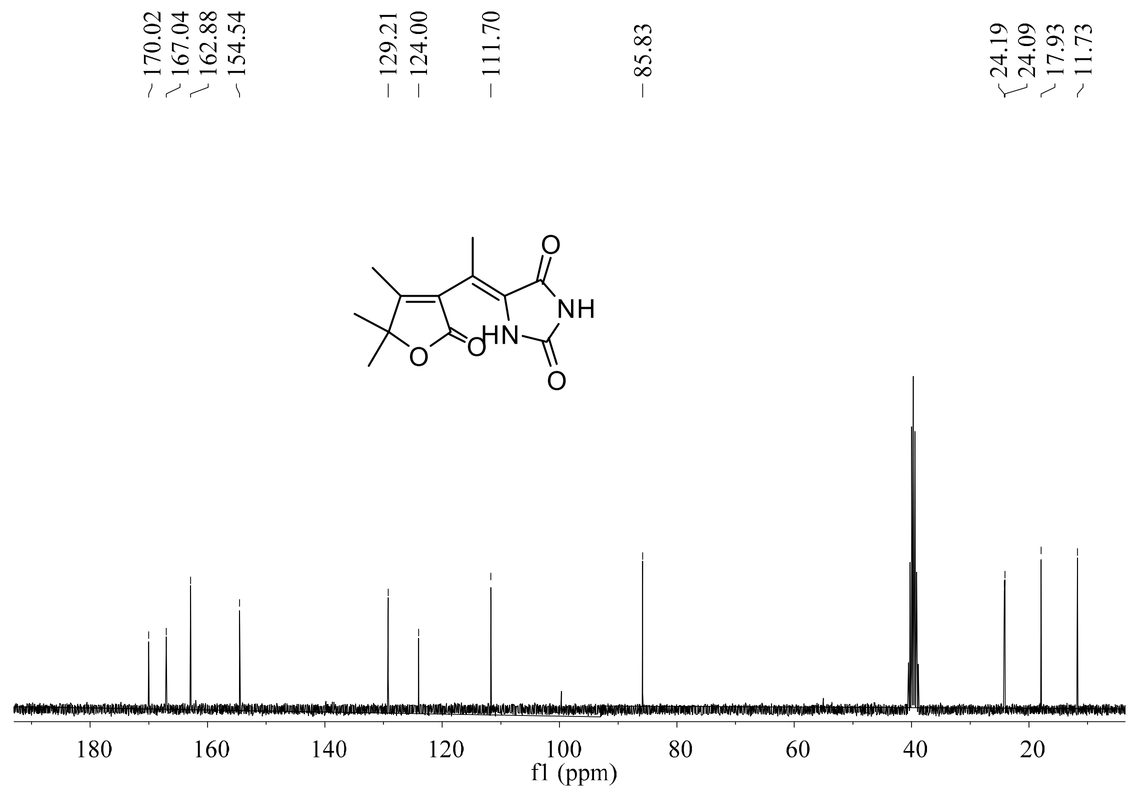

Figure S13. ^{13}C-NMR of **6**.

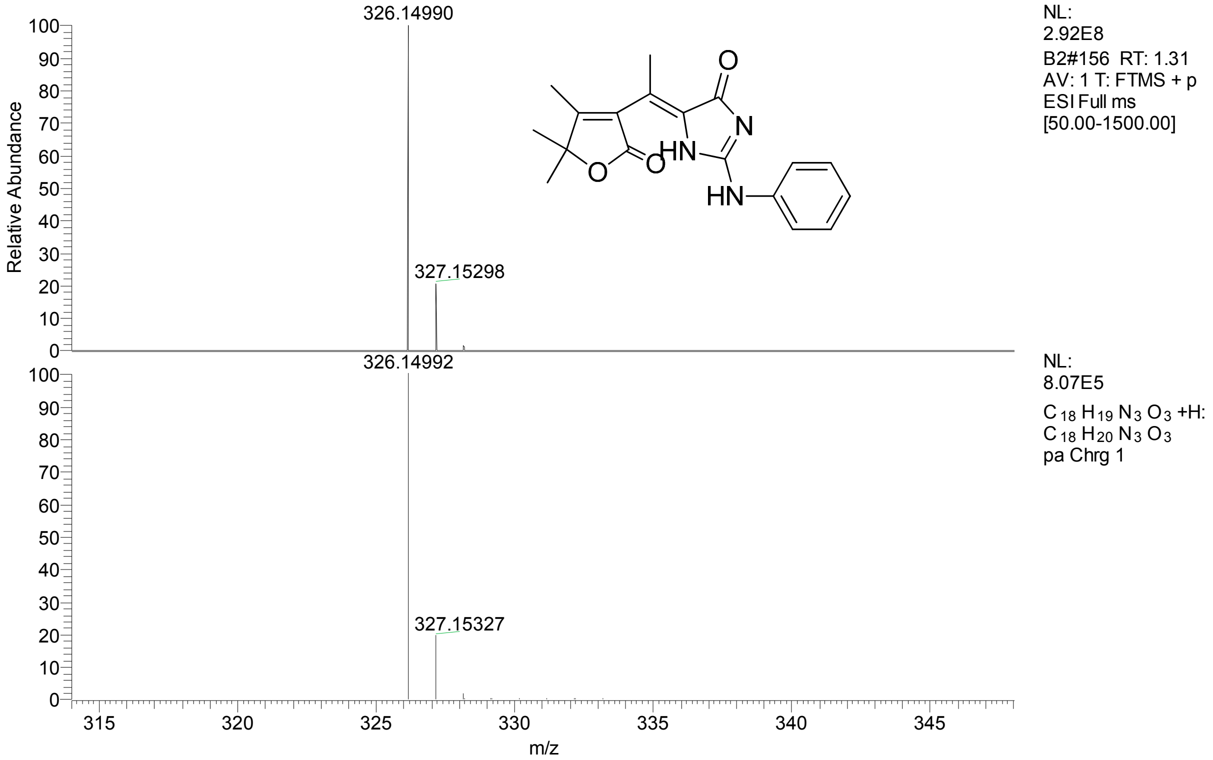

Figure S14. HR-ESI-MS of **4a**.

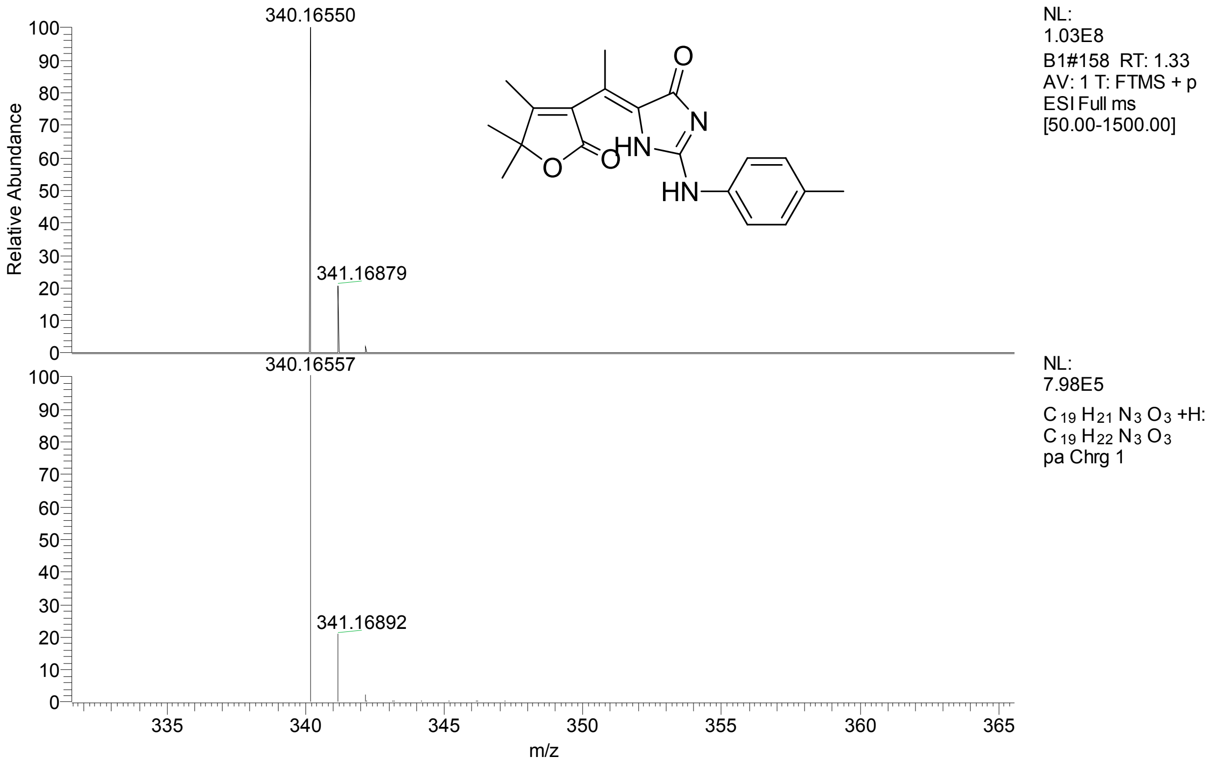

Figure S15. HR-ESI-MS of **4b**.

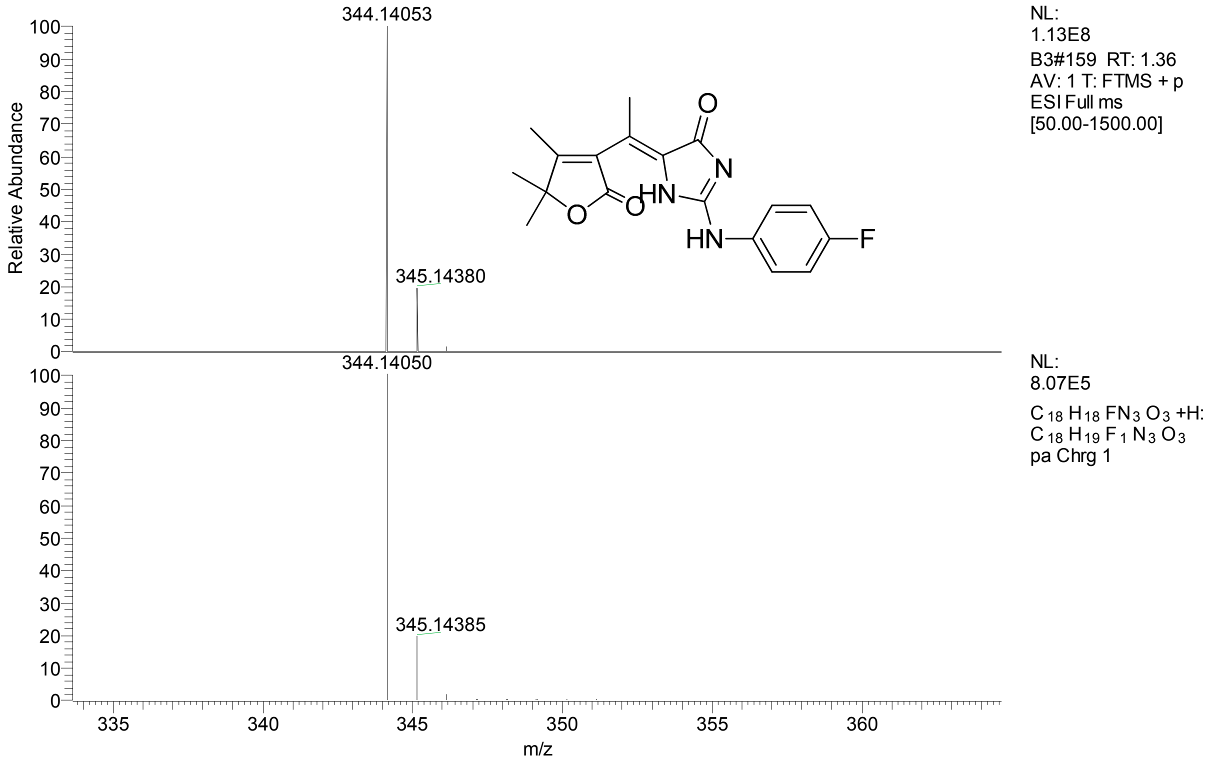

Figure S16. HR-ESI-MS of **4c**.

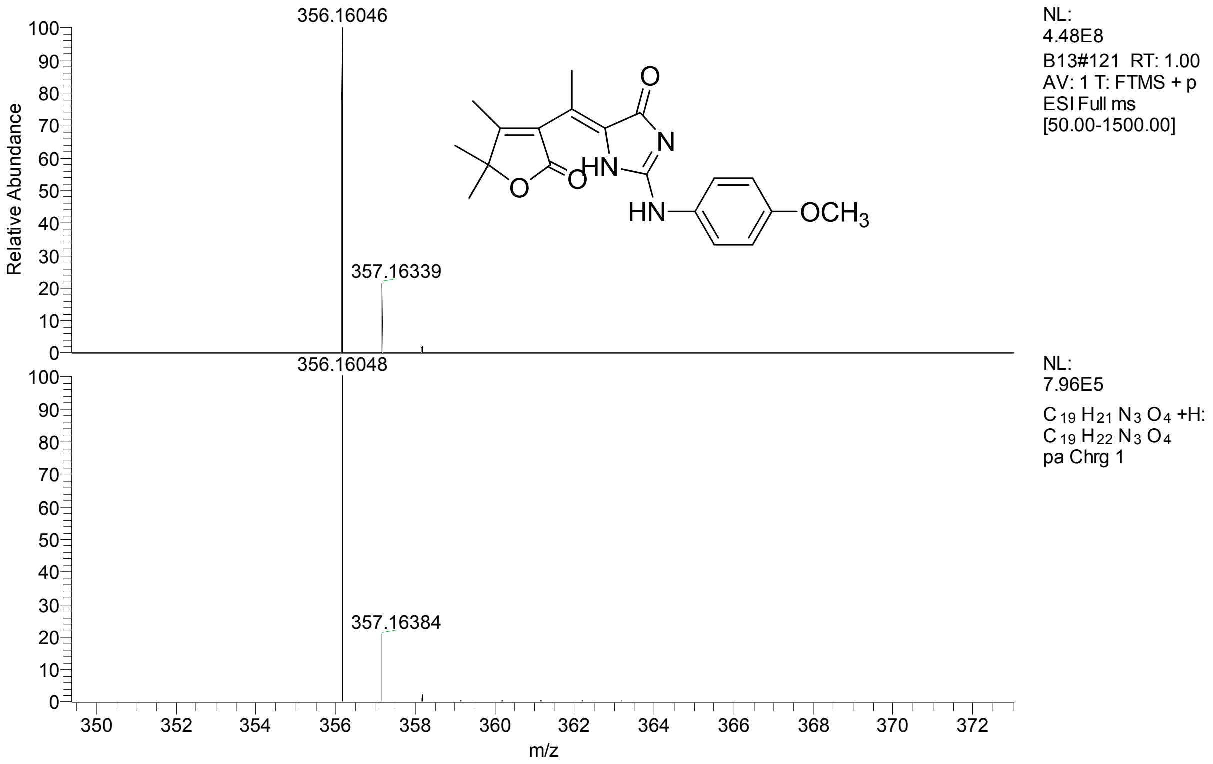

Figure S17. HR-ESI-MS of **4d**.

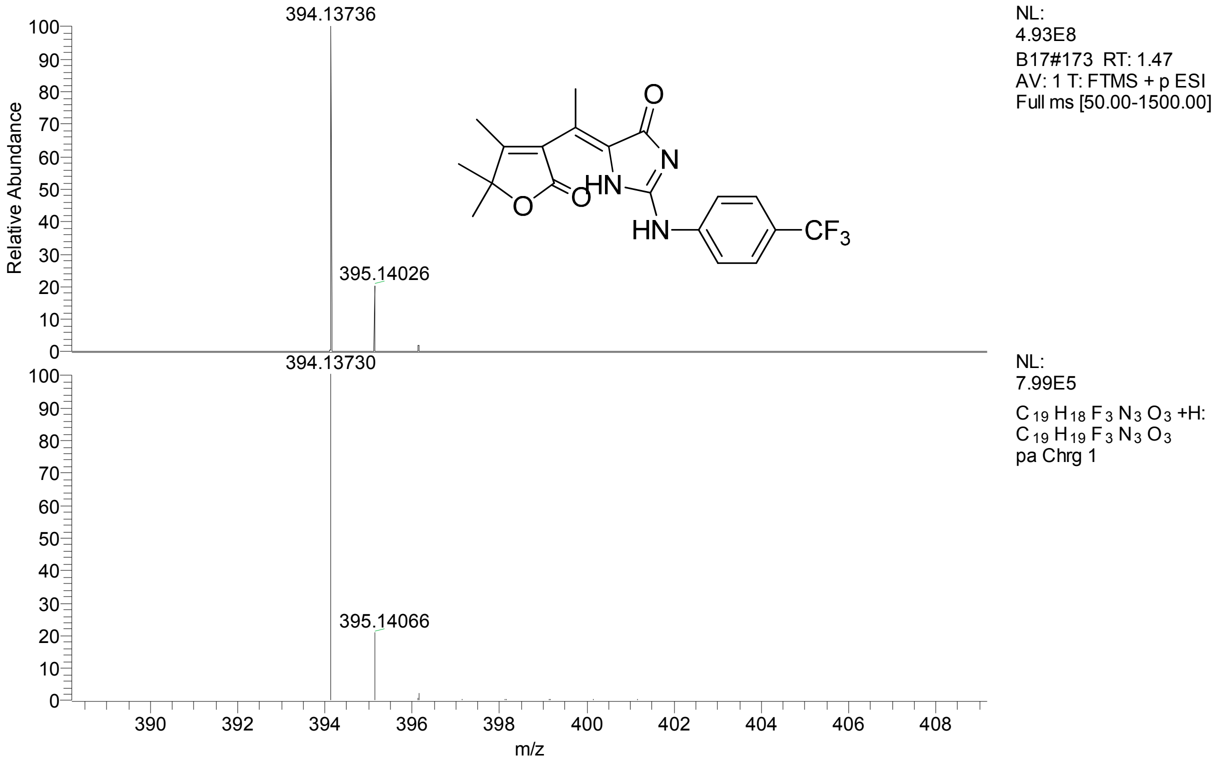

Figure S18. HR-ESI-MS of **4e**.

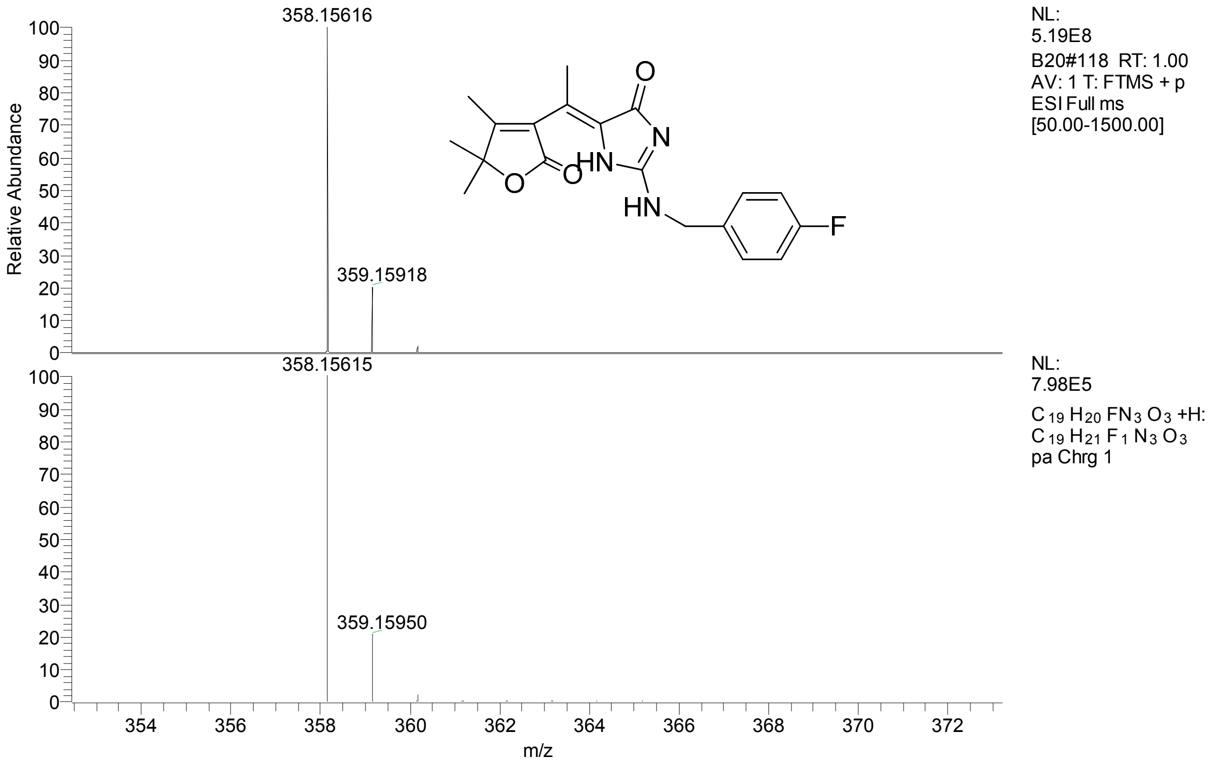

Figure S19. HR-ESI-MS of **4p**.

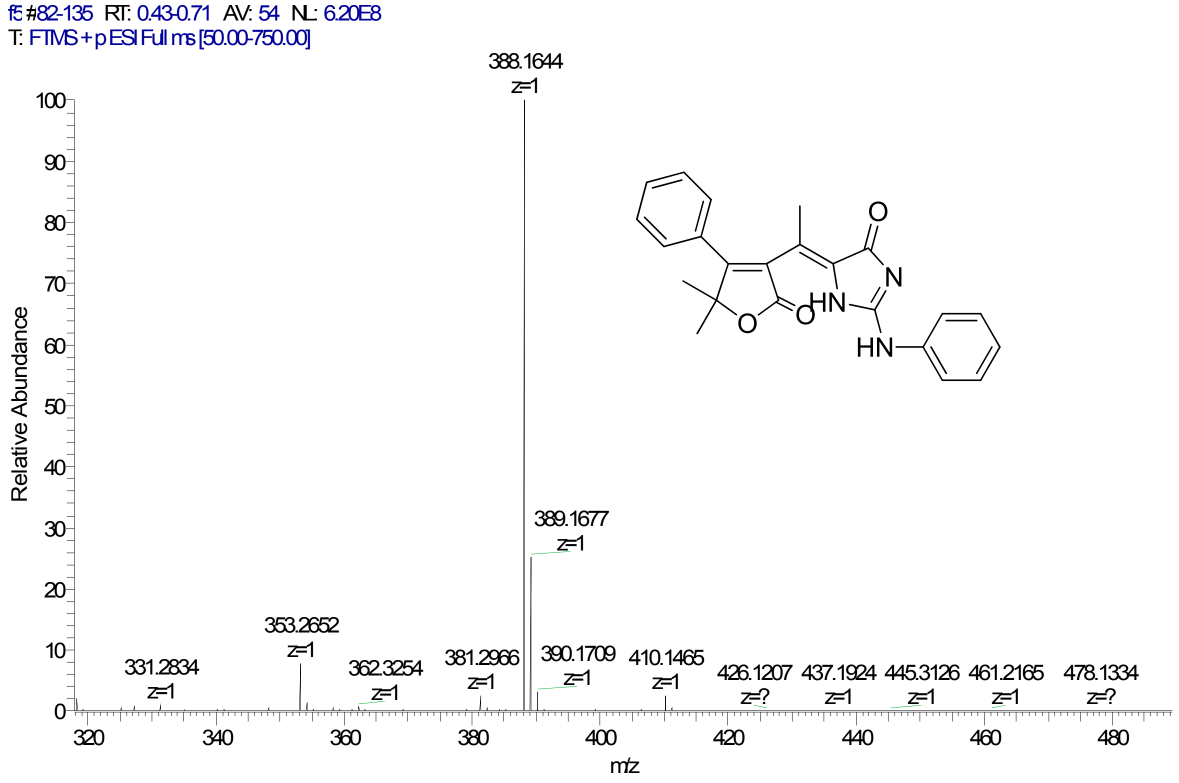

Figure S20. HR-ESI-MS of **5a**.

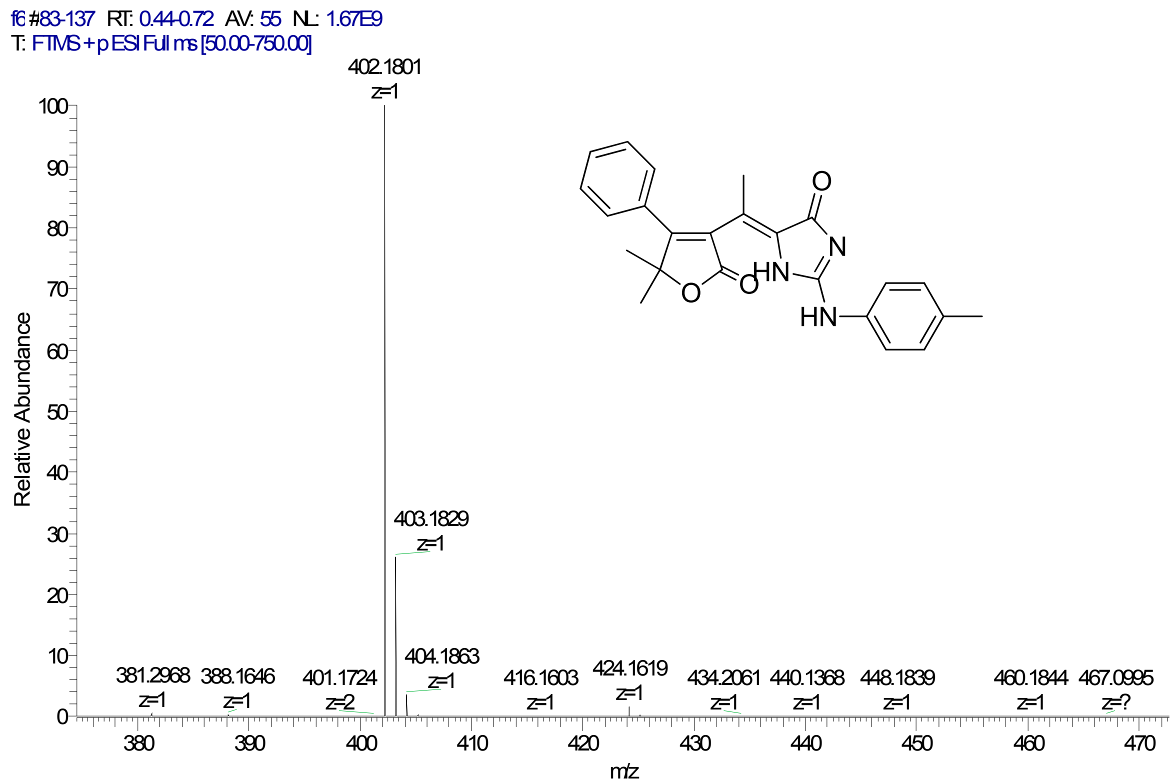

Figure S21. HR-ESI-MS of **5b**.

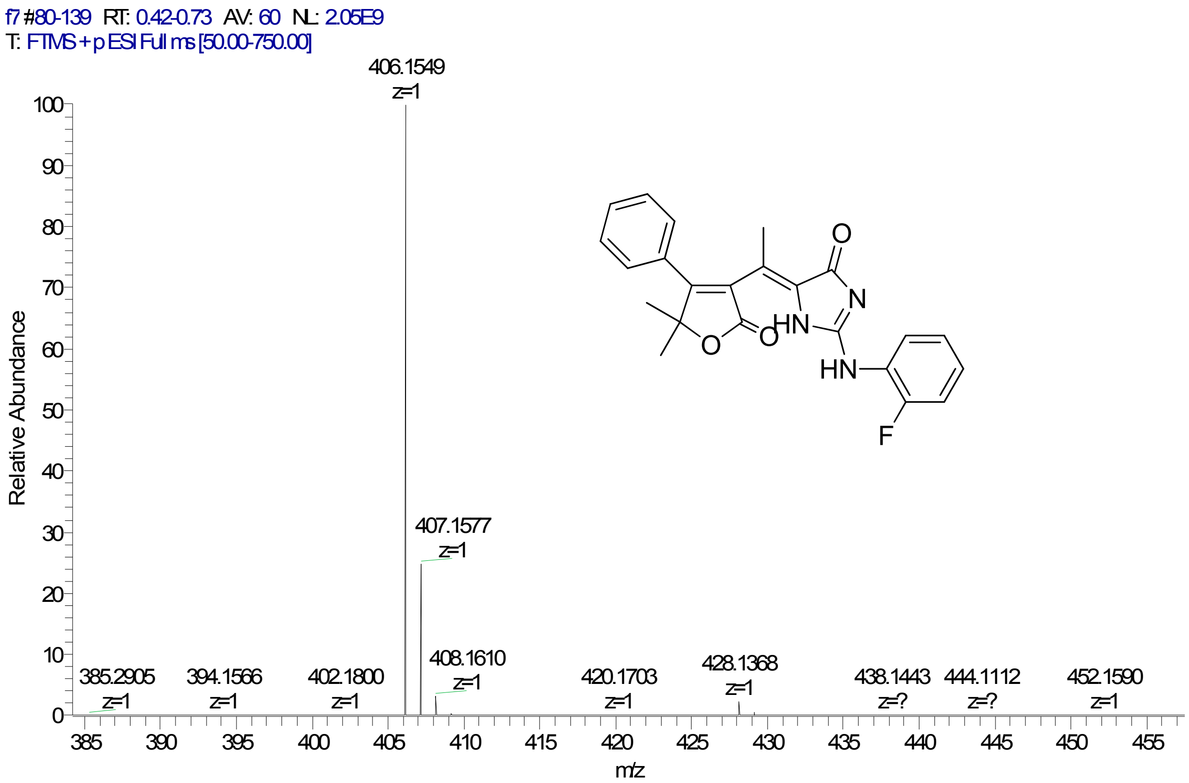

Figure S22. HR-ESI-MS of **5g**.

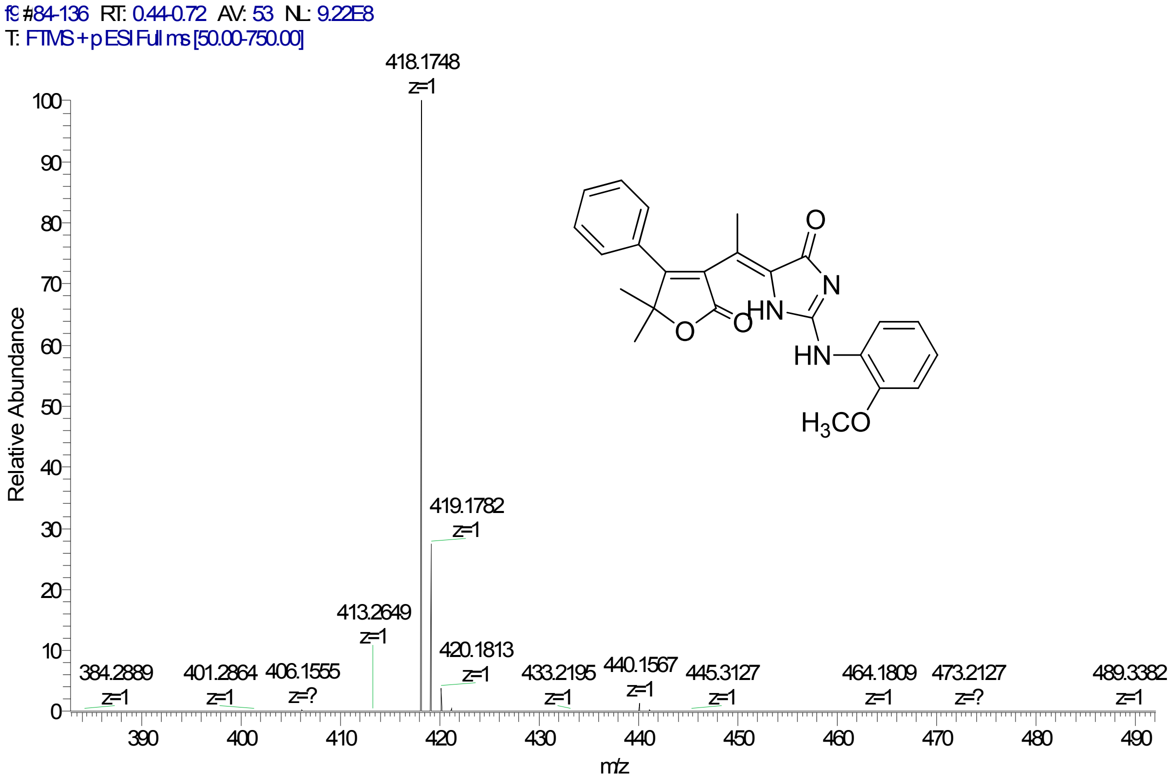

Figure S23. HR-ESI-MS of **5i**.
